# Revision of *Streblocera* Westwood (Hymenoptera, Braconidae, Euphorinae) from China, with the description of seven new species

**DOI:** 10.3897/zookeys.1044.59979

**Published:** 2021-06-16

**Authors:** Jun Li, Cornelis van Achterberg, Min-Lin Zheng, Jia-Hua Chen

**Affiliations:** 1 State Key Laboratory of Ecological Pest Control for Fujian and Taiwan Crops, Fuzhou, Fujian 35002, China; 2 Key lab of Biopesticide and Chemical Biology, Ministry of Education, Fujian Agriculture and Forestry University, Fuzhou, Fujian 35002, China; 3 Institute of Biological control, Fujian Agriculture and Forestry University, Fuzhou, Fujian 35002, China; 4 State Key Laboratory of Rice Biology, Ministry of Agriculture Key Lab of Agricultural Biology of Crop Pathogens and Insects, and Institute of Insect Sciences, Zhejiang University, Hangzhou 310058, China

**Keywords:** Townesilitini, taxonomy, identification, new records, key

## Abstract

The Chinese fauna of the euphorine genus *Streblocera* Westwood, 1833 (Hymenoptera, Braconidae) is revised. Seven new species from China are described and illustrated: Streblocera (Eutanycerus) carinifera Li, Chen and van Achterberg, **sp. nov**., S. (E.) laterostriata Li, Chen and van Achterberg, **sp. nov**., S. (E.) uncifera Li, Chen and van Achterberg, **sp. nov**., S. (S.) interrupta Li, Chen and van Achterberg, **sp. nov**., S. (S.) stigenbergae Li, Chen and van Achterberg, **sp. nov**., S. (S.) trullifera Li, Chen and van Achterberg, **sp. nov**., and S. (S.) zoroi Li, Chen and van Achterberg, **sp. nov**. An identification key to the females of *Streblocera* from China is provided.

## Introduction

Euphorinae (Hymenoptera, Braconidae) is a large subfamily of endoparasitoid wasps with more than 1,270 described species worldwide (Yu et al. 2016). In addition to the Aphidiinae, they are the only known Braconidae wasps attacking adult hosts. The most commonly used hosts are adult Coleoptera, especially of the families Chrysomelidae and Curculionidae, but adult Hymenoptera, Neuroptera, Hemiptera, Psocoptera and, rarely, Orthoptera are used (Shaw, 1985; Stigenberg et al. 2015). Obviously, coping with adult insect hosts was the major cause of the high diversity observed in adult Euphorinae.

Their special morphological structures are apparently modified for grasping more aggressive adult hosts during oviposition. One of the most bizarre modifications found in the Braconidae is in the females of *Streblocera*; here the antennae became raptorial. Typically, the scape has a unique horn, and one or more flagellomere are flattened and provided with claw-like structures. In many of the tropical species, the raptorial condition of the female antenna is accentuated by an unusual long scape (Shaw 1985).

The antennae of female euphorine genera *Betelgeuse*, *Marshiella*, *Streblocera*, and *Ropalophorus* are uniquely modified, and they are all koinobiont endoparasitoids of adult Coleoptera (but the host of *Betelgeuse* is yet unknown). In the most recent phylogeny by Stigenberg et al. (2015)*Streblocera* is included in the tribe Townesilitini, together with *Marshiella*, *Townesilitus*, and *Proclithrophorus*, while *Betelgeuse* is included in the Dinocampini and *Ropalophorus* in the Cosmophorini.

Chen and van Achterberg (1997) divided the genus *Streblocera* into five subgenera: *Asiastreblocera*, *Cosmophoridia*, *Eutanycerus*, *Villocera*, and *Streblocera*. However, specimens sometimes combine characteristics of different subgenera, especially of *Eutanycerus* and *Villocera* and both were synonymized by Belokobylskij (2000). Therefore, we consider the use of subgenera provisional without large-scale DNA-sequencing efforts and, for the moment, we use them for convenience.

Currently, 121 valid species of the genus Streblocera are known. To date, the subgenus Asiastreblocera comprises five species from the Oriental region and three species from the Palaearctic region; the subgenus Cosmophoridia comprises one species from the Oriental region and one species from the Palaearctic region; the subgenus Eutanycerus comprises 47 species from the Oriental region and ten species from the Palaearctic region; the subgenus Villocera comprises one species from the Oriental region and one species from the Palaearctic region; finally, the subgenus Streblocera comprises 18 species from the Oriental region and 12 species from the Palaearctic region (Yu et al. 2016; Long and Nhi 2020). From the Neotropical region only one described species is known, which belongs to the subgenus Lecythodella. It is characterised by having the malar suture deep and clearly defined, the dorsal carinae of first metasomal tergite absent, the third antennal segment shorter than the fourth segment and the occipital carina absent medio-dorsally. Stigenberg and Zhang (2020) included two species from New Guinea in *Streblocera* lacking the enlarged scapus (the scapus is much shorter than the third antennal segment) and having no dorsope in the first metasomal tergite. The enlarged scapus (longer than the first flagellomere and at least reaching upper level of vertex in lateral view, if intermediate then should have dorsope present) is generally used in the recognition of the genus, e.g. Shaw (1985), Chen and van Achterberg (1997), and Long and Pham (2020). Only the distinctly raised antennal sockets and molecular data hint to a relationship with *Streblocera* and, therefore, the inclusion of both species in *Streblocera* is questionable and likely both species belong to a new genus of the *Streblocera* clade.

## Materials and methods

Studied material was selected from the entomological collections of Biological Control Research Institute, Fujian Agriculture and Forestry University, Fuzhou, China (FAFU) (former Beneficial Insects Institute, China (BIIC)). The specimens were collected using a sweep net. All specimens studied are deposited in FAFU.

The specimens were examined using a Zeiss Stemi 2000 stereomicroscope. Photographs were taken with a Leica DFC450 digital camera mounted on a Leica M205C stereo microscope. All images were further processed using minor adjustment in Adobe Photoshop CC. Morphological terminology follows van Achterberg (1988, 1993), including the abbreviations for the wing venation. Sclerite surface sculpturing follows Eady (1968). Measurements are taken as indicated by van Achterberg (1988). For identification of the subfamilies, see van Achterberg (1993), for a key to the genus see Shaw (1985).

## Results and discussion

### Streblocera

Taxon classificationAnimaliaHymenopteraBraconidae

Westwood, 1833

75909605-9599-5323-8FDF-05FE22B63DC2

Figures 1 , 2 , 3 , 4 , 5 , 6 , 7 , 8 , 9 , 10 , 11 , 12 , 13 , 14 , 15 , 16 , 17 , 18 , 19 , 20 , 21

Streblocera Westwood, 1833: 342; Muesebeck 1936: 13; De Saeger 1946: 144; Shenefelt 1969: 125; S. Shaw 1985: 337; Tobias 1986: 235; Chou 1990: 91; Chao 1993: 61; Chen and van Achterberg 1997: 103; Stigenberg et al. 2015: 587. Type species (by monotypy): Streblocera fulviceps Westwood, 1833.
Eutanycerus Foerster, 1863: 251. Type species (by original designation): Eutanycerus halidayanus Foerster, 1863. Synonymised by Dalla Torre 1898.
Lecythodella Enderlein, 1912: 38–41. Synonymised by Muesebeck 1936.
Cosmophoridia Hedqvist, 1955: 93. Type species (by original designation): Cosmophorus flaviceps Marshall, 1897. Synonymised by Čapek and Snoflák 1959.
Streblocera subgenus Asiastreblocera Belokobylskij, 1987: 161; Chou 1990: 93; Chao 1993: 61. Type species (by original designation): Streblocera cornuta Chao, 1964.
Streblocera subgenus Villocera Chen and van Achterberg, 1997: 123. Type species (by original designation): Streblocera villosa Papp, 1985 (= Streblocera xianensis Wang, 1983). Synonymised by Belokobylskij 2000.

#### Diagnosis.

See Chen and van Achterberg (1997).

#### Distribution.

Afrotropical, Palaearctic, Oriental and Neotropical regions.

#### Biology.

Endoparasitoids of adult Chrysomelidae (*Chaetocnema cilindrica* (Baly); *Medythia nigrobilineata* Motschulsky; *Medythia suturalis* Motschulsky) (Yu et al. 2016).

**Figure 1.**
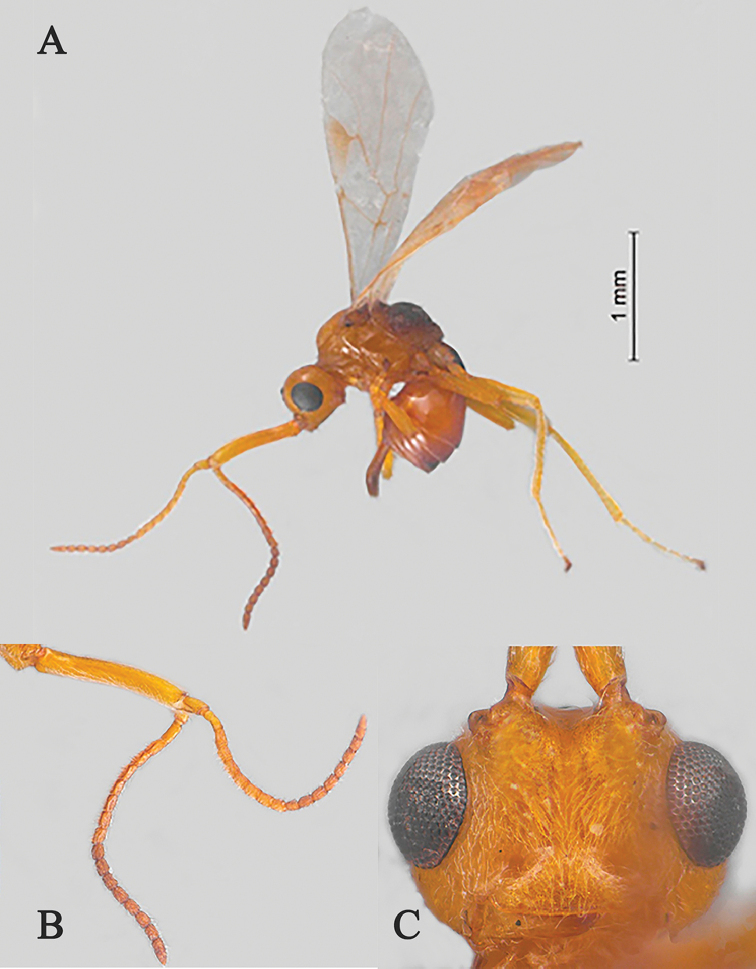
Streblocera (Cosmophoridia) flaviceps Marshall, 1898, ♀ **A** habitus, lateral aspect **B** antenna **C** head, anterior aspect.

### Key to females of the genus *Streblocera* from China

**Table:** 

1	Mandible with wide ventral lamella (Fig. 1C); inner concave side of scapus densely setose (Fig. 1B); Fujian, Zhejiang; (subgenus Cosmophoridia)	**S. (C.) flaviceps (Marshall, 1898)**
–	Mandible without wide ventral lamella (Figs 7E, 14G); inner concave side of scapus usually less densely setose (Figs 2C, 14C)	**2**
2	Face with an acute horn (Chen and van Achterberg 1997: fig. 49); fifth metasomal sternite with pair of acute teeth (Chen and van Achterberg 1997: fig. 49); (subgenus Asiastreblocera)	**3**
–	Face without an acute horn (Figs 7E, 14G); fifth metasomal sternite without acute teeth	**5**
3	Scape very robust, ca. 3.3× longer than its maximum width and with carina (Chen et al. 2004: fig. 612); first flagellomere long triangular, ca. 3.6× as long as wide, ca. 2.0× longer than second flagellomere (Chen et al. 2004: fig. 612); body yellow; body length 4.0 mm; Fujian	**S. (A.) planicornis (Chen and He, 2000)**
–	Scape slender, 6.2–7.0× longer than its maximum width and without carina (Chen et al. 2004: figs 610, 611); first flagellomere very long and with long acute apex (Chen et al. 2004: figs 610, 611)	**4**
4	Face with a shorter, simple median horn (Chen et al. 2004: figs 604, 610); antenna with 19 antennomeres; first flagellomere 0.5× as long as scapus (Chen et al. 2004: fig. 610) ; first metasomal tergite smooth (Chen et al. 2004: fig. 605); body yellowish brown; body length ca. 4.0 mm; Fujian, Zhejiang	**S. (A.) cornuta (Chao, 1964)**
–	Face with a longer horn, which has a medial carina (Wang 1983a: fig. 2); antenna with 17 antennomeres; first flagellomere 0.8× as long as scapus (Wang J-R 1983a: fig. 2); first metasomal tergite longitudinally and coarsely rugose (Wang 1983a: fig. 3); body yellowish brown; body length ca. 3.0 mm; Shaanxi, Taiwan, Zhejiang	**S. (A.) dayunesis (Wang, 1983)**
5	Face as long as wide, strongly flattened and tomentose (Fig. 29C); antennal sockets reaching up to upper level of eyes in lateral view (Fig. 29D); fifth flagellomere modified (Fig. 29B); Fujian, Guizhou, Taiwan, Zhejiang; (subgenus Villocera)	**S. (V.) villosa Papp, 1985**
–	Face longer than wide, more or less convex and at most densely setose (Figs 7E, 17D); antennal sockets near level of middle of eyes in lateral view (Fig. 10A); fifth flagellomere usually not modified	**6**
6	First flagellomere modified, with protruding corner apically, in typical species second flagellomere submedially inserted on first flagellomere, fifth to seventh flagellomeres normal (Fig. 16, 17B, 19, 20B, 23A, 21C, 24C, 27C); (subgenus Streblocera)	**7**
–	First flagellomere normal, without protruding corner apically, in typical species second flagellomere apically inserted on first flagellomere, first to seventh flagellomeres usually modified (Figs 2D, 4B, 5B, 6B, 7C, 9C, 10C, 11B, 12C, 13C, 14D); (subgenus Eutanycerus)	**27**
7	Basal half of scapus with distinct horn (Figs 20B, 24C, 27C); scapus robust, 1.4–3.0× longer than wide except S. (S.) fulviceps (Figs 20B, 24C, 27C); antenna with 17 or 18 antennomeres	**8**
–	Basal half of scapus without a distinct horn (Figs 17B, 23B, 21C); scapus slender, 4.4–8.5× longer than wide except S. (S.) emarginata (Figs 17B, 23B, 21C); antenna with 14–18 antennomeres	**14**
8	Basal half of scapus with two horns: lower horn twisted and upper one shark fin-shaped (Figs 27C, 28D); body yellowish brown to dark brown; body length ca. 2.6 mm; Hubei	**S. (S.) zoroi sp. nov.**
–	Basal half of scapus with one distinct horn (Figs 20B, 24C)	**9**
9	Scapus slender, not expanded, 4.5–9.0× longer than wide, with a distinct and broad horn near its base (Chen and van Achterberg 1997: fig. 535); occipital carina nearly complete (Chen et al. 2004: fig. 701); body length ca. 2.5 mm; Jilin	**S. (S.) fulviceps Westwood, 1833**
–	Scapus robust, expanded, 1.4–3.0× longer than wide; occipital carina interrupted medio-dorsally (Fig. 25D)	**10**
10	First flagellomere almost triangular (Fig. 20B); scapus 2.0–2.8× longer than wide (Fig. 2 in Chou 1990)	**11**
–	First flagellomere almost rectangular (Fig. 24C); scapus ca. 1.4 or 3.0× longer than wide (Fig. 24C; Chou 1990: fig. 4)	**12**
11	Scapus ca. 2.0× longer than wide (Chou 1990: fig. 2); first flagellomere less acute apically, ca. 1.5× longer than wide, ca. 1.5× longer than second flagellomere (Chou 1990: fig. 2); antenna with 18 antennomeres; body dark brown; body length ca. 2.8 mm; Taiwan	**S. (S.) triquetra Chou, 1990**
–	Scapus 2.7–2.8× longer than wide; first flagellomere more acute apically, 1.8–2.0× longer than wide, ca. 2.7× longer than second flagellomere (Fig. 20B); antenna with 17 antennomeres; body yellowish brown to reddish brown; body length 2.6–2.8 mm; Hubei	**S. (S.) spasskensis Belokobylskij, 2000**
12	Scapus ca. 1.4× longer than wide (Chou 1990: fig. 4); body dark brown; body length ca. 2.5 mm; Taiwan	**S. (S.) immensa Chou, 1990**
–	Scapus ca. 3.0× longer than wide (Fig. 24C)	**13**
13	Basal scapus with a spoon-shaped horn (Figs 24C, 25E); first flagellomere strongly expanded, ca. 1.6× longer than wide (Fig. 24C); vein SR1+3-SR of fore wing largely unsclerotized (Fig. 26J); antenna with 17 antennomeres; body yellowish brown to dark brown; body length ca. 2.5 mm; Liaoning	**S. (S.) trullifera sp. nov.**
–	One third of scapus with a wide horn (Chou 1990: fig. 5); first flagellomere strongly expanded, ca. 3.0× longer than wide (Chou 1990: fig.5); vein SR1+3-SR of fore wing sclerotized; antenna with 18 antennomeres; body dark brown; body length ca. 2.7 mm; Taiwan	**S. (S.) latibrocha Chou, 1990**
14	Basal half of scapus with a weak horn (Chou 1990: fig. 23) or carina (Fig. 17B)	**15**
–	Basal half of scapus without a horn or carina (Fig. 23B, 21C)	**17**
15	First flagellomere with a short hook apically (Chou 1990: fig. 13); first metasomal tergite 2.2–2.3× longer than its apical width (Chou 1990: fig. 264); antenna with 15 antennomeres; body yellowish brown to brown; body length 2.2–2.3 mm; Taiwan	**S. (S.) lini Chou, 1990**
–	First flagellomere with a long hook apically (Fig. 17B); first metasomal tergite ca. 1.9× longer than its apical width (Fig. 18H)	**16**
16	Occipital carina narrowly interrupted medio-dorsally and convex dorsally (You et al. 1993: fig. 6); scapus ca. 6.5× longer than its maximum width (You et al. 1993: fig. 9); inner side of eye straight in dorsal view (You et al. 1993: fig. 6); antenna with 17 antennomeres; body yellow; body length ca. 2.0 mm; Hunan	**S. (S.) hei You and Xiao, 1993**
–	Occipital carina nearly complete, very narrowly interrupted medio-dorsally and straight dorsally (Fig. 17C); scapus ca. 4.4× longer than its maximum width (Fig. 17B); inner side of eye curved in dorsal view (Fig. 17B); antenna with 15 antennomeres; body yellowish brown to dark brown; body length ca. 3.3 mm; Hubei	**S. (S.) interrupta sp. nov.**
17	Scapus strongly inflated, 2.1–2.6× longer than its maximum width (Chou 1990: fig. 2); first and second flagellomeres normally setose; first flagellomere shorter, 2.1–2.6× longer than wide (Chou 1990: fig. 2); body yellowish brown; body length 1.9–2.3 mm; Taiwan	**S. (S.) emarginata Chou, 1990**
–	Scapus slender, 3.6–9.0× longer than its maximum width (Fig. 21C; Chou 1990: fig. 10); first and second flagellomeres often densely setose (Fig. 21D); first flagellomere longer, 3.7–6.6× longer than wide (Fig. 21D; Chou 1990: fig.10)	**18**
18	First flagellomere longer, 5.1–6.6× longer than wide and 2.1–2.7× longer than second flagellomere (Fig. 21D)	**19**
–	First flagellomere shorter, 2.5–4.7× longer than wide and 1.3–2.0× longer than second flagellomere (Chou 1990: fig. 22)	**23**
19	Occipital carina interrupted medio-dorsally and convex dorsally (Fig. 22E); antenna with 15–16 antennomeres	**20**
–	Occipital carina complete (Chou 1990: fig. 87); antenna with 15–18 antennomeres	**22**
20	Occipital carina widely interrupted medio-dorsally (Belokobylskij, 2000: fig. 30); scapus more robust, 3.6–3.8× longer than its maximum width (Beolokobylskij, 2000: fig. 33); body brown; body length 2.0–2.2 mm; Fujian	**S. (S.) jezoensis Belokobylskij, 2000**
–	Occipital carina narrowly interrupted medio-dorsally (Fig. 22E); scapus slender, 5.2-8.5 × longer than its maximum width (Fig. 22E)	**21**
21	Antenna with 16 antennomeres; first flagellomere 6.1× longer than wide, with hook apically (Chou 1990: fig. 10); scutellar sulcus wide (Chou 1990: fig. 174); body brown; body length ca. 2.6 mm; Taiwan	**S. (S.) lalashanensis Chou**, **1990**
–	Antenna with 15 antennomeres; first flagellomere 5.1× longer than wide, without hook apically (Figs 21C, 21D); scutellar sulcus very wide (Fig. 21G); body yellowish brown to dark brown; body length ca. 2.2 mm; Yunnan	**S. (S.) stigenbergae sp. nov.**
22	Lower margin of clypeus without pair of tubercles; ovipositor almost straight (Chen et al. 2004: fig. 733); body reddish brown; body length ca. 3.0 mm; Fujian	**S. (S.) tachulaniana Chao**, **1964**
–	Lower margin of clypeus with pair of tubercles (Chou 1990: fig. 57); ovipositor curved; body brown; body length 3.1–3.3 mm	**S. (S.) meifengensis Chou, 1990**
23	First flagellomere strongly curved (Chou 1990: fig. 22)	**24**
–	Third flagellomere nearly straight or weakly curved (Chou 1990: figs 6, 7, 8, 11)	**25**
24	First metasomal tergite ca. 1.6× longer than as its apical width, distance across spiracles 0.9× distance from spiracle to apex (Chou 1990: fig. 266); antenna with 15 antennomeres; body yellowish brown; body length ca. 2.1 mm; Taiwan	**S. (S.) panda Chou, 1990**
–	First metasomal tergite ca. 2.0× longer than its apical width, distance across spiracles 1.1× distance from spiracle to apex (Chen et al. 2004: fig. 726); antenna with 16 antennomeres; body yellowish brown to reddish brown; body length ca. 3.0 mm; Fujian	**S. (S.) shaowuensis Chao, 1964**
25	Antenna with 17 antennomeres, combined length of second and following flagellomeres 1.8–1.9× longer than scapus (Chou 1990: figs 6, 21)	**26**
–	Antenna with 14–16 antennomeres, combined length of second and following flagellomeres 2.2–2.4× longer than scapus (Chou 1990: figs 7, 8)	**27**
26	Scapus slender, 7.8–8.0× longer than wide (Chou 1990: fig. 21); body yellowish brown; body length 2.4–2.6 mm; Taiwan	**S. (S.) chiuae Chou, 1990**
–	Scapus more robust, ca. 5.0× longer than wide (Chou 1990: fig. 6); body dark brown; body length ca. 2.8 mm; Taiwan	**S. (S.) tunpuensis Chou, 1990**
27	Scapus slender, 6.0–6.3× longer than wide (Chou 1990: fig. 8); body yellowish brown; body length 1.8–2.2 mm; Taiwan	**S. (S.) helvenaca Chou, 1990**
–	Scapus more robust, 4.4–5.1× longer than wide (Chou 1990: fig. 7); body dark brown; body length 2.4–2.5 mm; Fujian, Taiwan, Zhejiang	**S. (S.) tayulingensis Chou, 1990**
28	Flagellomeres not distinctly geniculate (Fig. 7C); body dark brown to black; Oriental region	**29**
–	Flagellomeres distinctly geniculate (Fig. 9C); colour of body variable; Palaearctic and Oriental regions	**32**
29	First to seventh flagellomeres without small prominence (Fig. 7C); occipital carina nearly complete, interrupted medio-dorsally (Fig. 7D)	**30**
–	First to seventh flagellomeres with small prominence (Chen and van Achterberg 1997: fig. 507); occipital carina complete (Chen and van Achterberg 1997: fig. 508)	**31**
30	Antenna with 21 antennomeres, scapus ca. 5.7× as long as maximum wide (Chen et al. 2004: fig. 693); first metasomal tergite ca. 2.5× longer than its apical width, smooth laterally (Chen et al. 2004: fig. 695); propodeum with basal carina; ovipositor curved apically; body black; body length ca. 2.7 mm; Sichuan	**S. (E.) sichuanensis Wang, 1986**
–	Antenna with 24 antennomeres, scapus ca. 7.5× as long as maximum wide (Fig. 7C); first metasomal tergite ca. 2.0× longer than its apical width, striate laterally (Fig. 8H); propodeum without basal carina (Fig. 8F); ovipositor straight (Fig. 7B); body dark brown to black; body length ca. 4.0 mm; Yunnan	**S. (E.) laterostriata sp. nov.**
31	First flagellomere 3.0–3.4× longer than wide (Chou 1990: fig. 29); first metasomal tergite 2.8–3.1× longer than its apical width, smooth (Chou 1990: fig. 252); body dark brown; body length 2.9–3.0 mm; Taiwan	**S. (E.) primotina Chou, 1990**
–	First flagellomere ca. 2.0× longer than wide (Chen and van Achterberg 1997: fig. 507); first metasomal tergite2.3× longer than its apical width, longitudinally striate (Chen and van Achterberg 1997: fig. 510); body dark brown; body length ca. 3.1 mm; Fujian	**S. (E.) linearata Chen and van Achterberg, 1997**
32	Flagellomeres geniculate at fifth or sixth flagellomere (Fig. 2D; Chou 1990: fig. 20); occipital carina nearly complete, interrupted medio-dorsally (Fig. 2E)	**33**
–	Flagellomeres geniculate at seventh or eighth flagellomere (Fig. 4B; Chou 1990: fig. 2); occipital carina complete or nearly complete	**35**
33	Scapus with a pair of spines apically (Wang, 1983b: figs 3, 4); first metasomal tergite more robust, ca. 1.6× longer than its apical width (Wang J-R, 1983b: fig. 7); propodeum with rather long basal carina; femur with dark brown ring basally; body yellowish brown; body length ca. 3.0 mm; Shaanxi	**S. (E.) xianensis Wang, 1983**
–	Scapus without spines apically; first metasomal tergite slender, 2.1–2.6× longer than its apical width (Fig. 3J); propodeum with rather long or short basal carina (Fig. 3I; Chou 1990: fig. 224); femur without dark brown ring basally	**34**
34	Fourth flagellomere without hook (Chou 1990: fig. 26); scapus 5.7× longer than its maximum width, without horn (Chou 1990: fig. 26); first metasomal tergite more robust, 2.1–2.3× longer than its apical width, largely smooth (Chou 1990: fig. 253); body yellowish brown to dark brown; body length 3.3–4.5 mm; Taiwan	**S. (E.) quinaria Chou, 1990**
–	Fourth flagellomere with hook (Fig. 2D); scapus 9.3× longer than its maximum width, with small horn (Fig. 2C); first metasomal tergite slender, ca. 2.6× longer than its apical width, smooth basally, striate laterally (Fig. 3J); body yellowish brown to brown; body length ca. 3.1 mm; Fujian	**S. (E.) carinifera sp. nov.**
35	Flagellomeres geniculate at eighth flagellomere (Chou 1990: fig. 25; Chao, 1993: fig. 2); scapus without horn basally (Chou 1990: fig. 25; Chao, 1993: fig. 2)	**36**
–	Flagellomeres geniculate at seventh flagellomere (Figs 4B, 5B, 6B, 9C, 10B, 11B, 12C, 13C, 14D); scapus without horn or with horn basally	**37**
36	Eighth flagellomere without prominence (Chou 1990: fig. 25); scapus 8.4–9.0× longer than its maximum width (Chou 1990: fig. 25); occipital carina nearly complete, interrupted medio-dorsally (Chou 1990: fig. 71); body yellowish brown; body length 2.3–2.9 mm; Taiwan	**S. (E.) octava Chou, 1990**
–	Eighth flagellomere with hook (Chao, 1993: fig. 2); scapus 7.0× longer than its maximum width (Chao, 1993: fig. 2); occipital carina complete; body brown; body length ca. 4.2 mm; Fujian	**S. (E.) ekphora Chao, 1993**
37	Scapus with horn basally, 5.4–9.3× longer than wide (Figs 4B, 5A, 6A, 9B, 10B, 12B, 13B, 14C); first metasomal tergite more robust, 1.7–2.3× longer than its apical width; propodeum with basal carina (Chen and van Achterberg 1997: fig. 502) or without basal carina (Chou 1990: fig. 220)	**38**
–	Scapus without horn basally, 7.1–9.4× longer than wide (Fig. 11A); first metasomal tergite slender, 2.3–3.1× longer than its apical width; propodeum with basal carina (Chou 1990: figs 211, 215)	**54**
38	First to seventh flagellomeres serrate ventrally (Figs 6B, 14D); antenna with 23–24 antennomeres	**39**
–	First to seventh flagellomeres straight ventrally (Figs 4B, 5B, 6B, 9C, 10C, 12C, 13C); antenna with 12–32 antennomeres	**40**
39	First to seventh flagellomeres serrate ventrally and all with hook (Chen et al. 2004: fig. 660); first metasomal tergite ca. 2.3× longer than its apical width; body dark brown; body length ca. 3.8 mm; Fujian, Shaanxi	**S. (E.) hsiufui You, 1999**
–	First to seventh flagellomeres serrate ventrally, only the carina of seventh flagellomere with hook (Fig. 14D); first metasomal tergite ca. 1.7× longer than its apical width (Fig. 12K); body yellowish brown to brown (Fig. 14A); body length ca. 4.4 mm; Yunnan	**S. (E.) uncifera sp. nov.**
40	Sixth flagellomere more or less with hook, not firmly fused to seventh flagellomeres (Figs 9C, 10C, 13C)	**41**
–	Sixth flagellomere without hook, firmly fused to seventh flagellomeres (Fig. 12C)	**50**
41	Scapus very long, ca. 9.8× longer than wide (Chou 1990: fig. 27); occipital carina complete (Chou 1990: fig. 64); antenna with 20 antennomeres; body dark brown; body length 6.0–6.5 mm; Taiwan	**S. (E.) amplissima Chou, 1990**
–	Scapus shorter, 6.0–8.5× longer than wide (Chou 1990: figs 26, 30); occipital carina complete or interrupted medio-dorsally; antenna with 19–26 antennomeres	**42**
42	Combined length of eighth flagellomere and following flagellomeres shorter than scapus (Figs 9A, 13A); occipital carina interrupted medio-dorsally (Chou 1990: figs 68, 78); propodeum with short basal carina (Chou 1990: figs 216, 227); antenna with 19–20 antennomeres	**43**
–	Combined length of eighth flagellomere and following flagellomeres longer than scapus (Fig. 10A); occipital carina complete or interrupted medio-dorsally; propodeum with basal carina or without basal carina; antenna with 21–26 antennomeres	**44**
43	Scapus 7.2–8.5× longer than wide (Fig. 9B); propodeum without distinct median area (Chou 1990: fig. 216); antenna with 20 antennomeres; body yellowish brown; body length ca. 4.3 mm; Fujian, Hubei, Jilin, Taiwan, Yunnan	**S. (E.) lienhuachihensis Chou, 1990**
–	Scapus ca. 6.0× longer than wide (Fig. 13B); propodeum with distinct median area (Chou 1990: fig. 227); antenna with 19–20 antennomeres; body yellowish brown; body length 3.3–3.5 mm; Fujian, Hubei, Taiwan	**S. (E.) tsuifengensis Chou, 1990**
44	Antenna with 19–22 antennomeres (Fig. 10A); propodeum with rather short basal carina (Chou 1990: fig. 220) or without basal carina (Chou 1990: fig. 221); body yellowish brown; body length 2.4–2.6 mm; Anhui, Fujian, Guizhou, Hebei, Henan, Hubei, Hunan, Jiangsu, Jiangxi, Jilin, Liaoning, Qinghai, Shaanxi, Shanxi, Shandong, Taiwan, Yunnan, Zhejiang	**S. (E.) okadai Watanabe, 1942**
–	Antenna with 24–29 antennomeres; propodeum with basal carina (Chen and van Achterberg 1997: fig. 502)	**45**
45	First to seventh flagellomeres rather narrow, first flagellomere without sensillae (Chou 1990: fig. 28); body dark brown; body length ca. 3.8 mm; Taiwan	**S. (E.) destituta Chou, 1990**
–	First to seventh flagellomeres wider, first flagellomere with 4–7 sensillae	**46**
46	At basal 0.4 of scapus with a horn at inner side and scapus 6.0–6.9× longer than wide (Chen and van Achterberg 1997: fig. 513; Chen et al. 2002: fig. 90)	**47**
–	At basal 0.3 of scapus with a horn ventrally and scapus ca. 7.1× longer than wide	**48**
47	Scapus with a weak horn (Chen and van Achterberg 1997: fig. 513); first flagellomere longer, 1.3× longer than wide (Chen and van Achterberg 1997: fig. 513); body yellowish brown; body length 3.7–3.9 mm; Guizhou	**S. (E.) janus Chen and van Achterberg, 1997**
–	Scapus with a stronger horn (Chen et al. 2002: fig. 90); first flagellomere shorter, 2.5× longer than wide (Chen et al. 2002: fig. 90); body yellow to yellowish brown; body length ca. 4.8 mm; Guizhou	**S. (E.) liboensis Chen and He, 2002**
48	Scapus with a weak horn (Chen and van Achterberg 1997: fig. 528); seventh flagellomere with a long, distinct horn (Chen and van Achterberg 1997: fig. 528); body dark brown; body length 3.4–3.7 mm; Zhejiang	**S. (E.) cornis Chen and van Achterberg, 1997**
–	Scapus with a much stronger horn (Wang J-Y, 1981: fig. 1; Chou 1990: fig. 31); seventh flagellomere with a weak horn (Wang J-Y, 1981: fig. 1; Chou 1990: fig. 31); body length 4.8–6.0 mm	**49**
49	First flagellomere with 5–7 sensillae (Wang J-Y, 1981: fig. 1); body dark brown; body length 5.0–6.0 mm; Sichuan, Zhejiang	**S. (E.) emeiensis Wang, 1981**
–	First flagellomere with 4 sensillae (Chou 1990: fig. 31); body yellowish brown; body length ca. 4.8 mm; Taiwan	**S. (E.) taiwanensis Chou, 1990**
50	Scapus 9.0–9.9× longer than wide (Chou 1990: fig. 29); body yellowish brown; body length 4.3–4.9 mm; Taiwan	**S. (E.) nantouensis Chou, 1990**
–	Scapus 5.0–8.0× longer than wide (Figs 4B, 12B); body length 2.7–3.8 mm except S. (E.) gigantea	**51**
51	Scapus with a narrow and acute horn, 7.3–8.0× longer than wide (Fig. 12B); body brown; body length ca. 3.8 mm; Jilin, Taiwan	**S. (E.) sungkangensis Chou, 1990**
–	Scapus with a wide horn (but narrow in *S. gigantea*), 5.0–5.3× longer than wide (Fig. 4B)	**52**
52	Body length ca. 6 mm; antenna with 24–29 antennomeres; scapus with a narrow horn (Chen and van Achterberg 1997: fig. 518); body reddish brown; Fujian	**S. (E.) gigantea Chen and van Achterberg, 1997**
–	Body length 2.7–3.0 mm; antenna with 24–29 antennomeres; scapus with a wide horn (Chen and van Achterberg 1997: fig. 498; You and Xiao 1993: fig. 3)	**53**
53	Eighth flagellomere with horn (Chen and van Achterberg 1997: fig. 498); scapus ca. 5.0× longer than wide (Chen and van Achterberg 1997: fig. 498); antenna with 22 antennomeres; body reddish brown; body length ca. 2.7 mm; Zhejiang	**S. (E.) obtusa Chen and van Achterberg, 1997**
–	Eighth flagellomere without horn (You and Xiao 1993: fig. 3); scapus ca. 5.2× longer than wide (You and Xiao 1993: fig. 3); antenna with 23 antennomeres; body yellow; body length ca. 3.0 mm; Hubei, Hunan	**S. (E.) chaoi You and Zhou, 1993**
54	Scapus more robust, ca. 4.0× longer than wide, with carina apically (Chou 1990: fig. 28); antenna with 31–32 antennomeres; body black; body length 4.0–4.7 mm; Taiwan	**S. (E.) nigra Chou, 1990**
–	Scapus slender, 7.5–9.8× longer than wide, without carina apically (Chou 1990: figs 21–23); antenna with 21–24 antennomeres; body length 3.0–3.5 mm except S. (E.) distincta	**55**
55	First to seventh flagellomeres serrate ventrally (Chou 1990: fig. 23; Chen and van Achterberg 1997: 522)	**56**
–	First to seventh flagellomeres straight ventrally (Chou 1990: figs 7, 21, 22)	**57**
56	Antenna with 21–22 antennomeres; scapus ca. 9.8× longer than wide (Chou 1990: fig. 23); basal flagellomeres slightly serrate (Chou 1990: fig. 23); first metasomal tergite distinctly striate (Chou 1990: fig. 245); body black; body length 3.1–3.2 mm; Taiwan	**S. (E.) kenchingi Chou, 1990**
–	Antenna with 24 antennomeres; scapus ca. 7.1× longer than wide (Chen and van Achterberg 1997: fig. 522); basal flagellomeres deeply serrate (Chen and van Achterberg 1997: fig. 522); first metasomal tergite nearly smooth; body yellowish brown; body length 4.2–4.5 mm; Zhejiang	**S. (E.) distincta Chen and van Achterberg, 1997**
57	Lateral lobes of mesoscutum densely setose anteriorly (Chou 1990: fig. 163); scapus 7.5–7.8× longer than wide (Chou 1990: fig. 22); body dark brown; body length 2.9–3.0 mm; Fujian, Taiwan	**S. (E.) opima Chou, 1990**
–	Lateral lobes of mesoscutum only with very few setae along its anterior margin (Chen and van Achterberg 1997: fig. 253); scapus 8.0–9.4× longer than wide; body length 3.0–3.5 mm	**58**
58	Body dark brown; first metasomal tergite 2.7–2.9× longer than its apical width; scapus 9.2–9.4× longer than wide (Chou 1990: fig. 21); body dark brown; body length 3.4–3.5 mm; Taiwan	**S. (E.) adusta Chou, 1990**
–	Body yellowish brown; first metasomal tergite ca. 2.4× longer than its apical width; scapus ca. 8.0× longer than wide (You et al. 1988: fig. 7); body yellowish brown; body length 3.0–3.2 mm; Guangxi	**S. (E.) guangxiensis You and Zhou, 1988**

### Streblocera (Cosmophoridia) flaviceps

Taxon classificationAnimaliaHymenopteraBraconidae

Marshall, 1898

3845A558-F42A-591E-8451-47539F10C891

Figure 1A–C

Cosmophorus flaviceps Marshall, 1898: 208; Shenefelt 1969: 127; Tobias 1986: 237.
Cosmophoridia flaviceps ; Hedqvist, 1955: 237.
Streblocera (Cosmophoridia) flaviceps ; Belokobylskij, 1987: 162; Chen and van Achterberg 1997: 105; Belokobylskij 2000: 295.

#### Material.

1♀, C China, Hubei Province, Shennongjia, Hongping, 1.viii.1988, Jiangquan Yang, average altitude 2000 m; 1♀, NE China, Heilongjiang Province, Heihe city, Wudalianchi, 14.viii.2012, Yingying Zhao, average altitude 300 m; 1♀, NE China, Inner Mongolia Province, Wulanzuozhi, 15.viii.2011, Yingying Zhao, average altitude 1500 m; 1♀, SE China, Fujian Province, Mt Wuyi, Dazhulan, 23.vii.1986, Jiang, average altitude 1500 m.

#### Biology.

Unknown, but attracted to light (Papp and Rezbanyai-Reser 1996).

#### Distribution.

Oriental: China (Fujian and Zhejiang) and Palaearctic: Austria, China (Hubei, Heilongjiang, Inner Mongolia), Czech Republic, Germany, Japan, Korea, Russia and Switzerland.

### Streblocera (Eutanycerus) carinifera

Taxon classificationAnimaliaHymenopteraBraconidae

Li, Chen & van Achterberg sp. nov.

F694AA2B-83FD-5AF6-A38B-847F385D4A7D

http://zoobank.org/77D24161-4ADE-45A7-80A9-0989A9B892CD

Figure 2A–E , 3G–K

#### Type material.

Holotype, ♀, SE China, Fujian Province, Mt Wuyi, Guadang, 13.viii.1988, Jian-hua Ge, average altitude 1800 m. Paratype: 1♀, SE China, Fujian Province, Mt Wuyi, Xianfengling, 8.viii.1988, Jian-wen Chen, average altitude 1400 m.

#### Description.

**Figure 2.**
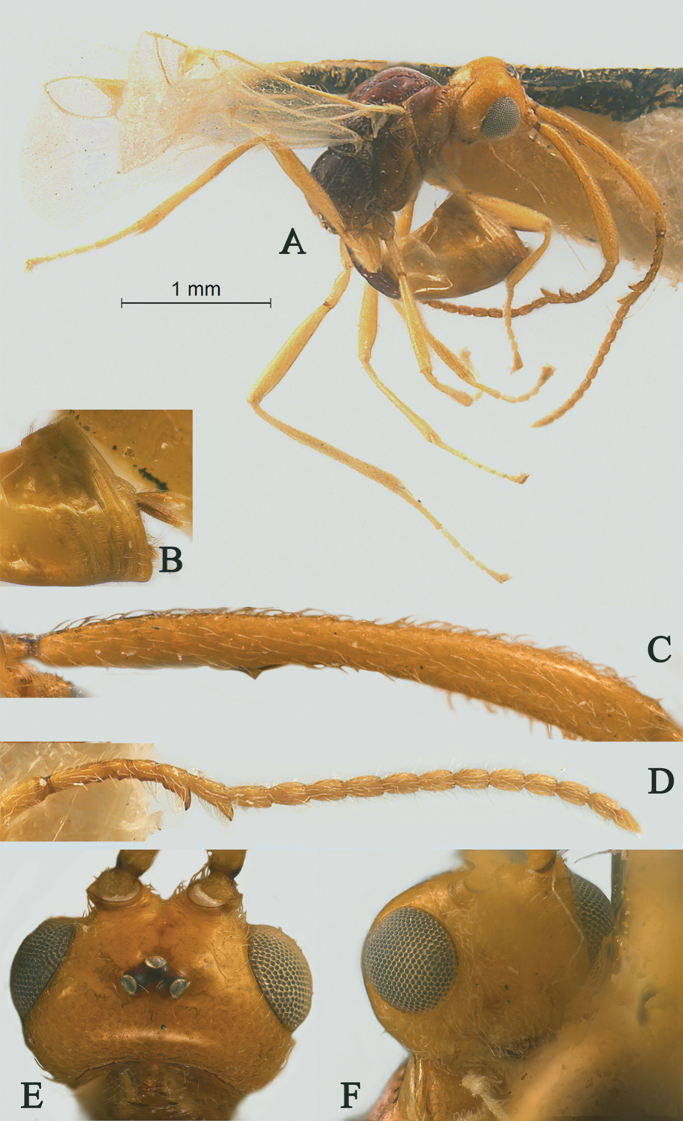
Streblocera (Eutanycerus) carinifera Li, Chen and van Achterberg, sp. nov., ♀ **A** habitus, lateral aspect **B** ovipositor and its sheaths, lateral aspect **C** scapus **D** flagellomeres, lateral view **E** head, dorsal aspect **F** head, anterior aspect.

Holotype, ♀, length of antenna 2.8 mm, of fore wing 2.1 mm, and of body 3.1 mm (Fig. 2A).

*Head.* Antenna with 18 antennomeres and 1.3× longer than fore wing, 0.9× as long as body (Fig. 2A); scapus rather long and slender and expanded, 9.3× longer than its maximum width, evenly curved, with a very small tooth-shaped horn, finely setose (Fig. 2C); first to fifth flagellomeres modified: flagellomeres geniculated at fifth flagellomere, first to fourth flagellomeres fused, first to fifth flagellomeres with carina ventrally, fourth and fifth flagellomeres with hook and, hook of fourth flagellomere larger than hook of fifth (Fig. 2D); first flagellomere 2.0× longer than second flagellomere, first, second and penultimate flagellomere 3.2, 1.5 and 1.8× longer than wide, respectively (Fig. 2D); eye 1.7× longer than temple in dorsal view (Fig. 2E); temples roundly behind eyes (Fig. 2E); ocelli medium-sized, OOL:OD:POL = 71:24:41 (Fig. 2E); frons and vertex largely smooth (Fig. 2E); occipital carina nearly complete, interrupted medio-dorsally (Fig. 2E); face 1.4× wider than high, smooth (Fig. 2F); clypeus smooth, narrow than face, convex, 2.0× wider than high (Fig. 2F); dorsal margin of clypeus under level of ventral margin of eye anterior (Fig. 2F); tentorial pits large (Fig. 2F); malar suture shallow and narrow, length of malar space 1.2× basal width of mandible (Fig. 2F); mandibles long and stout.

**Figure 3.**
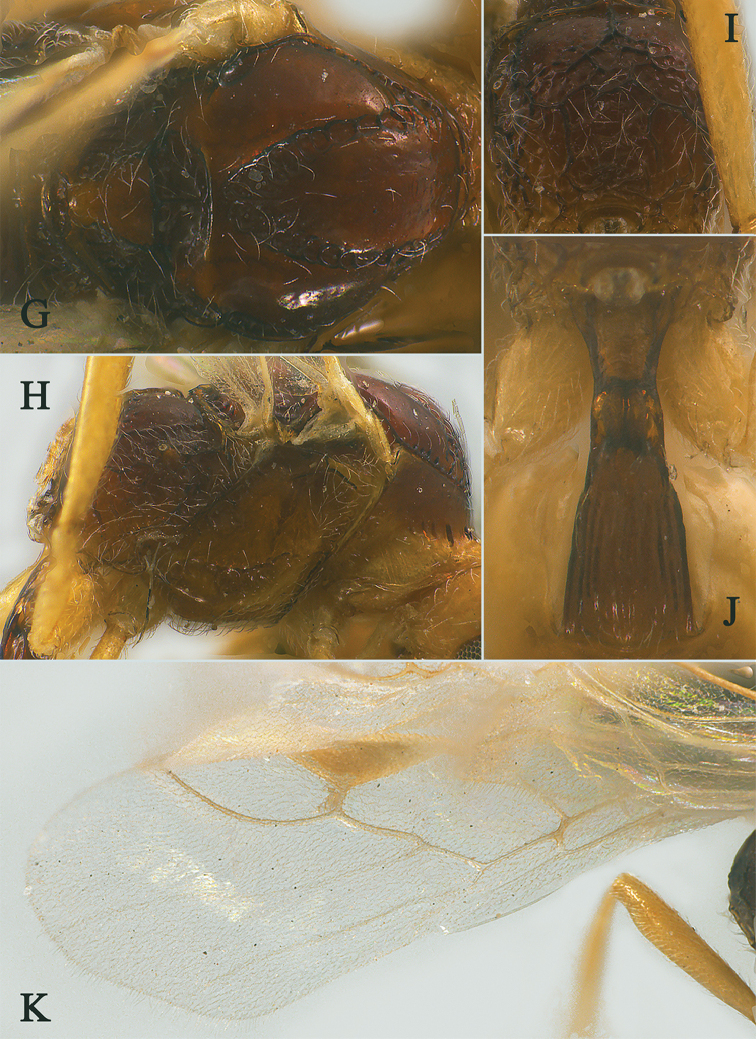
Streblocera (Eutanycerus) carinifera Li, Chen and van Achterberg, sp. nov., ♀ **G** mesosoma, dorsal aspect **H** mesosoma, lateral aspect **I** propodeum, dorsal aspect **J** first metasomal tergites, dorsal aspect **K** fore wing.

*Mesosoma.* Length of mesosoma 2.4× its height (Fig. 3H); side of pronotum crenulated anteriorly, largely smooth (Fig. 3H); propleuron smooth and shiny (Fig. 3H); mesopleuron smooth (Fig. 3H); prepectal medio-ventral carina present (Fig. 3H); episternal scrobe short and wide (Fig. 3H); precoxal sulcus long, wide and crenulate (Fig. 3H); mesonotum moderately sparsely setose, flat, smooth and shiny (Fig. 3G); notauli narrow and carina; mesoscutum sparsely setose, flattened (Fig. 3G); scutellar sulcus wide and rugose with one distinct crenula (Fig. 3G); scutellum flat, smooth (Fig. 3G); metapleuron reticulate (Fig. 3H); propodeum with short basal carina but not median area, largely rugulose (Fig. 3I).

*Wings.* Fore wing (Fig. 3K): vein 1-SR+M absent; vein 1-R1 0.7× as long as pterostigma; vein SR1+3-SR curved; r:2-SR = 17:58; vein r issued behind middle of pterostigma; vein m-cu cross vein 2-SR; vein cu-a slightly short than vein 1-CU1 and postfurcal.

*Legs.* Fore leg modified: tibia strong curved, 1.2× longer than coxa, 0.8× as long as femur, and femur 3.1× longer than wide; middle leg: tibia 5.2× longer than coxa, 1.2× longer than femur; hind leg: tibia 3.5× longer than coxa, 1.4× longer than femur; hind coxa smooth, 1.2× longer than wide; femur, tibia and basitarsus 7.7, 15.6 and 6.0× longer than wide, respectively; hind basitarsus 0.3× as long as hind tibia, and 0.6× as long as combined second to fifth tarsal segments; hind fourth tarsal segment 0.8× as long as fifth tarsal segment.

*Metasoma.* First tergite quiet slender, 2.6× longer than its apical width, apical width 1.9× its minimum width, with large dorsope basally (Fig. 3J); first tergite smooth basally, striate laterally (Fig. 3J); following tergites smooth and shiny; ovipositor sheath short and robust, 0.1× as long as fore wing; ovipositor short and robust (Fig. 2B).

*Colour.* Yellowish brown to brown; palpi pale yellow; legs yellowish brown; face, antenna, ovipositor sheaths and ovipositor yellowish brown; wing membrane hyaline, pterostigma and veins brown; mesosoma and first metasomal tergite brown.

#### Remarks.

This new species is similar to S. (E.) thayi Belokobylskij, 2000 from Vietnam, but differs from it as follows: (i) 18 antennomeres, scapus 9.3× longer than its maximum width, first to fifth flagellomeres with carina ventrally, fourth and fifth flagellomeres with hook and hook of fourth flagellomere larger than hook of fifth flagellomere (19 antennomeres, scapus 7.5× longer than its maximum width, first to fifth flagellomeres with carina ventrally, only fifth flagellomeres with hook in *S. thayi*); (ii) first metasomal tergite smooth basally, striate laterally (first tergite entirely and densely striate); (iii) ovipositor sheath shorter, 0.1× as long as fore wing (ovipositor sheath longer, 0.2× as long as fore wing); (iv) body reddish brown (body yellowish brown).

#### Biology.

Unknown.

#### Distribution.

Oriental: China (Fujian).

#### Etymology.

Named after the ventral carina of the first to fifth flagellomeres: *carina* means keel in Latin and *fero* is Latin for to carry.

### Streblocera (Eutanycerus) chaoi

Taxon classificationAnimaliaHymenopteraBraconidae

You, 1999

46BD1E63-15DD-590C-9F68-DF210F3EF10D

Figure 4A–B

Streblocera chaoi You and Zhou, 1993: 485; Chen and van Achterberg 1997: 109.

#### Material.

4♀, C China, Hubei Province, Shennongjia, Hongping, 11. viii. 1988, Jianquan Yang, average altitude 2000m; 1♀, same label data, but 16. viii. 1988; 1♀, C China, Hubei Province, Shennongjia, Honghua, 4. viii. 1988, Juchang Huang, average altitude 1800m; 2♀, C China, Hubei Province, Shennongjia, Muyu, 9. viii. 1988, Juchang Huang, average altitude 1200m.

#### Biology.

Unknown.

#### Distribution.

Oriental: China (Hunan) and Palaearctic: China (Hubei). New record for Palaearctic region.

**Figure 4.**
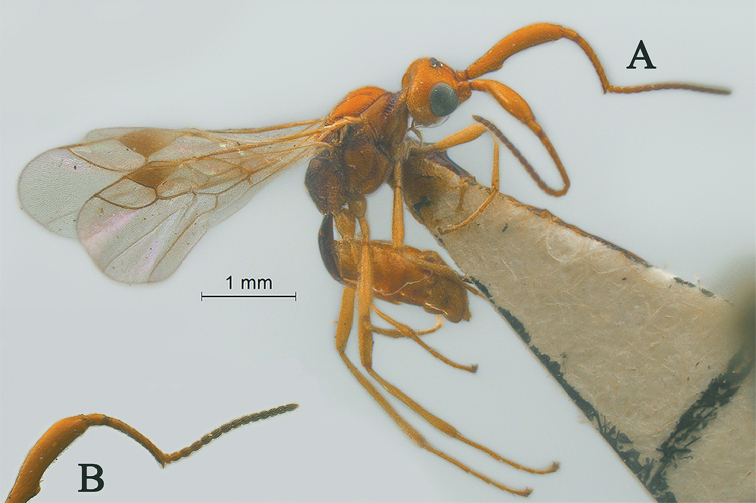
Streblocera (Eutanycerus) chaoi You, 1999, ♀ **A** habitus, lateral aspect **B** antenna.

### Streblocera (Eutanycerus) cornis

Taxon classificationAnimaliaHymenopteraBraconidae

Chen and van Achterberg, 1997

DC299405-B66C-5387-B381-562A8C516C46

Figure 5A–B

Streblocera (Eutanycerus) cornis Chen and van Achterberg, 1997: 109.

#### Material.

1♀, C China, Hubei Province, Shennongjia, Muyu, 5. viii. 1988, Li-qin Zhang, average altitude 1200m; 2♀, same label data, but 9. viii. 1988; 1♀, same label data, but 8. viii. 1988, Juchang Huang; 1♀, same label data, but 9. viii. 1988; 1♀, C China, Hubei Province, Shennongjia, Hongping, 14. viii. 1988, Jian-quan Yang, average altitude 2000m; 1♀, same label data, but 16. viii. 1988, Li-qin Zhang; 2♀, same label data, but 18. viii. 1988, Juchang Huang.

#### Biology.

Unknown.

#### Distribution.

Oriental: China (Guizhou and Zhejiang) and Palaearctic: China (Hubei). New record for Palaearctic region.

**Figure 5.**
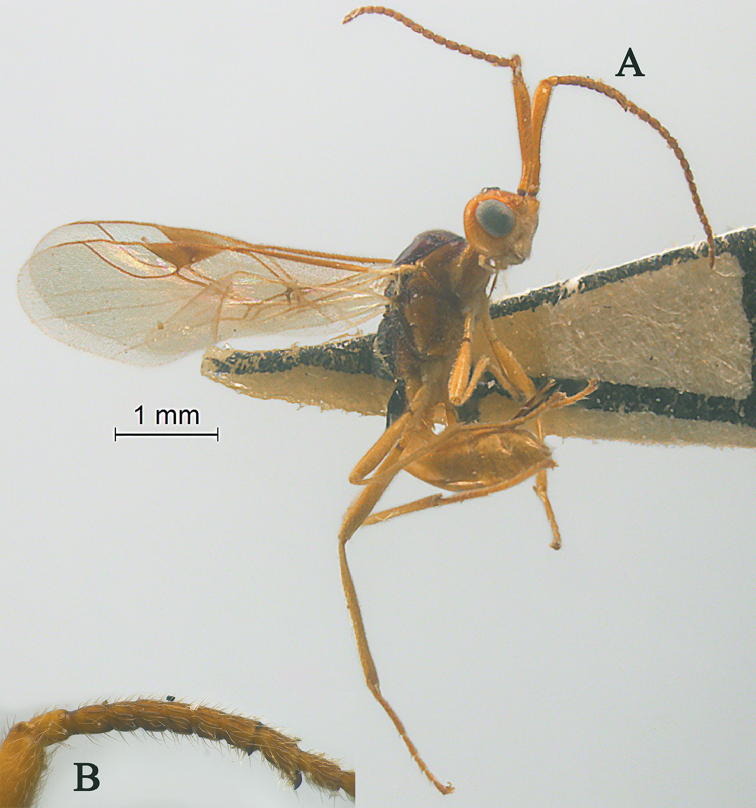
Streblocera (Eutanycerus) cornis Chen and van Achterberg, 1997, ♀ **A** habitus, lateral aspect **B** first to seventh flagellomeres.

### Streblocera (Eutanycerus) hsiufui

Taxon classificationAnimaliaHymenopteraBraconidae

You, 1999

E4AEAE7B-6642-5466-8DC1-5BB7EEBD4D71

Figure 6A, B

Streblocera (Cosmophoridia) serrata Chao, 1993: 65. Preoccupied by S. serrata Granger, 1949.
Streblocera (Eutanycerus) chaoi Chen and van Achterberg, 1997: 109. Nomen novum for S. serrata Chao, 1993; preoccupied by S. chaoi You and Zhou, 1993.
Streblocera hsiufui You, 1999: 54. Nomen novum for S. (E.) chaoi Chen and van Achterberg, 1997.
Streblocera (Eutanycerus) austrochinensis Belokobylskij, 2000: 278. Nomen novum for S. serrata Chao, 1993.

#### Material.

1♀, SE China, Fujian Province, Mt Meihua, 22. iii. 1986, Bao-bin Guan, average altitude 900m; 1♀, SE China, Fujian Province, Mt Wuyi, Sangan, 8. viii. 1988, Xiao-bin Zhang, average altitude 900m; 1♀, SE China, Fujian Province, Sanming city, Minjiangyuan, Yingtaoling, vegetable garden, 31. v. 2017, Lingfei Peng, average altitude 950m; 1♀, SE China, Fujian Province, Mt Wuyi (28°00N, 117°48E), 24. iv. 2015, Jun Li, average altitude 258m; 1♀, NW China, Shaanxi Province, Baoji city, Feng county, 3. iv. 1988, Jiangquan Yang, average altitude 1800m.

#### Biology.

Unknown.

#### Distribution.

Oriental: China (Fujian) and Palaearctic: China (Shaanxi). New record for Palaearctic region.

**Figure 6.**
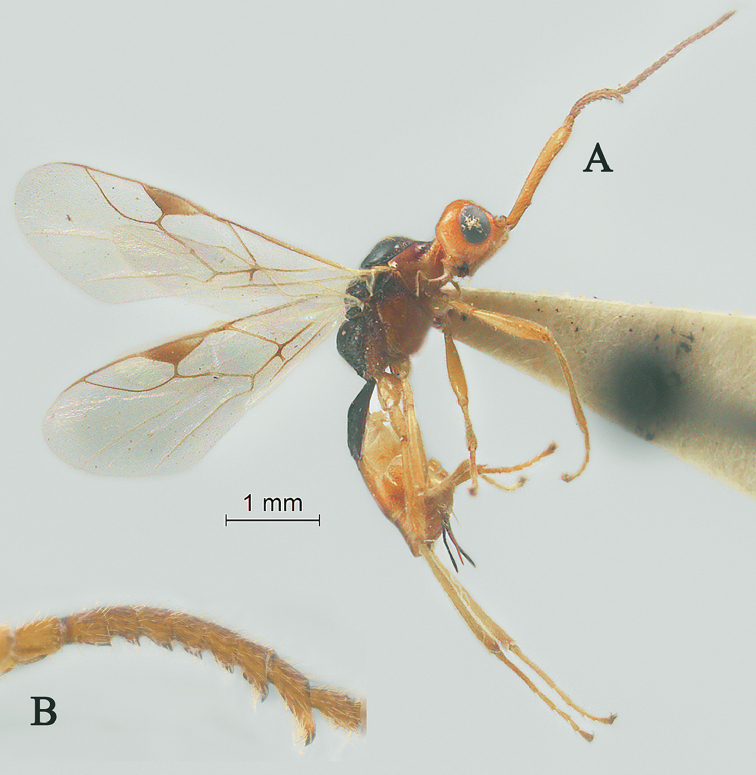
Streblocera (Eutanycerus) hsiufui You, 1999, ♀ **A** habitus, lateral aspect **B** first to seventh flagellomeres.

### Streblocera (Eutanycerus) laterostriata

Taxon classificationAnimaliaHymenopteraBraconidae

Li, Chen & van Achterberg sp. nov.

B9CC8BFC-3534-53DF-AC9A-F89DF9807793

http://zoobank.org/97A7D15E-CC23-4F72-9118-E5D6EC53CE9F

Figures 7A–E , 8F–I

#### Type material.

Holotype, ♀, SW China, Yunnan Province, Wenshan City, Malipo County, Tiechangtuanxiang, 20.viii.2017, Yan-Qiong Peng, 1372 m.

#### Description.

**Figure 7.**
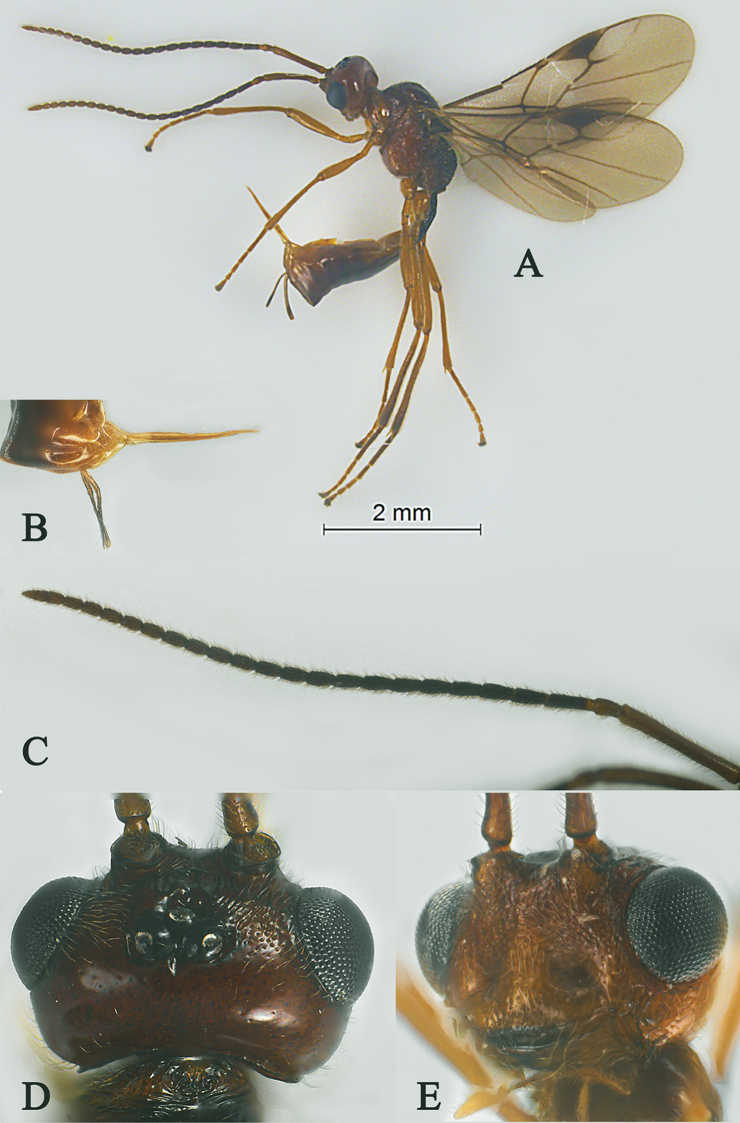
Streblocera (Eutanycerus) laterostriata Li, Chen and van Achterberg, sp. nov., ♀ **A** habitus, lateral aspect **B** ovipositor and its sheaths, lateral aspect **C** antenna **D** head, dorsal aspect **E** head, anterior aspect.

Holotype, ♀, length of antenna 3.9 mm, of fore wing 3.3 mm, and of body 4.0 mm (Fig. 7A).

*Head.* Antenna with 24 antennomeres and 1.2× longer than fore wing, 0.9× as long as body (Fig. 7A); scapus slender, straight and no expanded, 7.5× longer than its maximum width, without horn, finely setose (Fig. 7C); all flagellomeres unmodified, first flagellomere 1.5× longer than second flagellomere, first, second and penultimate flagellomere 3.5, 2.3 and 1.5× longer than wide, respectively (Fig. 7C); eye 1.5× longer than temple in dorsal view (Fig. 7D); temples roundly behind eyes ; ocelli medium size, OOL:OD:POL = 19:2:12 (Fig. 7D); frons rugose and setose (Fig. 7D); vertex largely punctate and setose (Fig. 7D); occipital carina nearly complete, interrupted medio-dorsally (Fig. 7D); face 2.6× wider than high, rugose to punctate (Fig. 7E); clypeus punctulate and convex, narrow slightly than face, 2.7× wider than high (Fig. 7E); dorsal margin of clypeus above level of ventral margin of eye in anterior view (Fig. 7E); tentorial pits very large (Fig. 7E); malar suture shallow and wide, length of malar space 0.7× basal width of mandible (Fig. 7E); mandibles stout (Fig. 7E).

**Figure 8.**
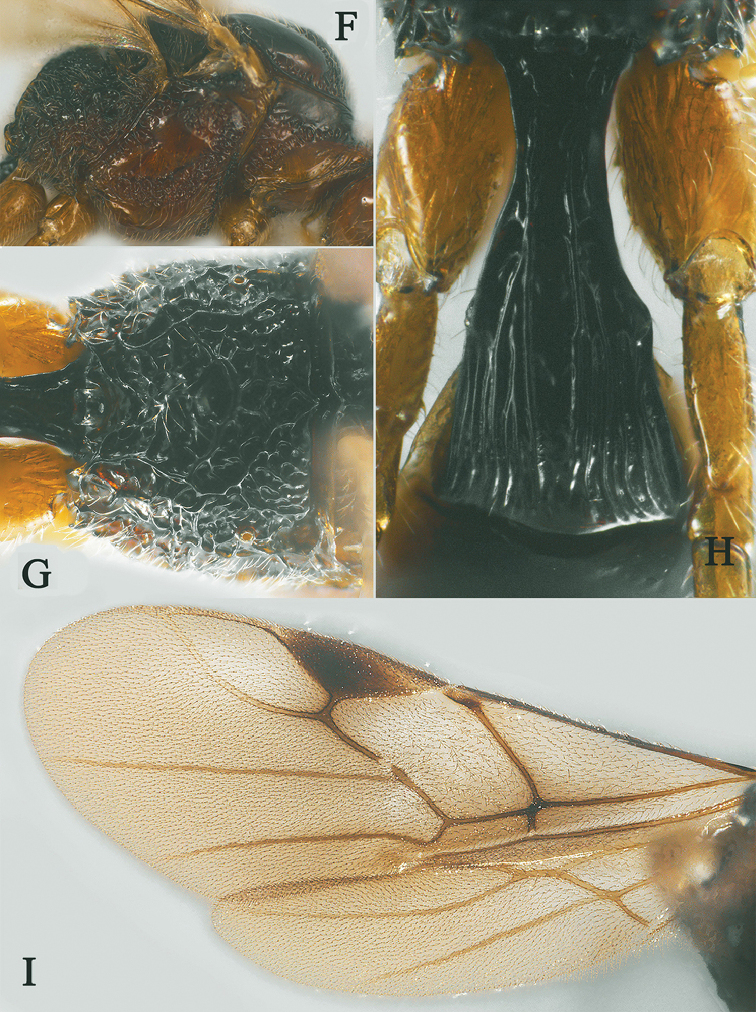
Streblocera (Eutanycerus) laterostriata Li, Chen and van Achterberg, sp. nov., ♀ **F** mesosoma, lateral aspect **G** propodeum, dorsal aspect **H** first metasomal tergites, dorsal aspect **I** wings.

*Mesosoma.* Length of mesosoma 1.6× its height (Fig. 8G); side of pronotum crenulated to rugose (Fig. 8G); propleuron smooth (Fig. 8G); mesopleuron rugose and foveolate (Fig. 8G); prepectal medio-ventral carina present (Fig. 8G); episternal scrobe wide (Fig. 8G); precoxal sulcus long, wide and crenulate (Fig. 8G); mesonotum sparsely setose, flat, smooth and shiny; notauli narrow and carina; mesoscutum sparsely setose, flattened; scutellar sulcus wide and rugose with one distinct crenula; scutellum flat, smooth; metapleuron reticulate (Fig. 8G); propodeum rugae and foveolae (Fig. 8F).

*Wings.* Fore wing (Fig. 8I): vein 1-SR+M absent; vein 1-R1 0.7× as long as pterostigma; vein SR1+3-SR curved, largely unstained; r:2-SR = 13:65; vein r issued slightly behind middle of pterostigma; vein m-cu cross vein 2-SR; vein cu-a distinct longer than vein 1-CU1 and postfurcal.

*Legs.* Fore leg: tibia 5.0× longer than coxa, 1.8× longer than femur; middle leg: tibia 4.4× longer than coxa, 1.3× longer than femur; hind leg: tibia 5.6× longer than coxa, 1.1× longer than femur; hind coxa smooth, 1.8× longer than wide; femur, tibia and basitarsus 6.1, 15.6 and 6.7× longer than wide, respectively; hind basitarsus 0.1× as long as hind tibia; hind fourth tarsal segment 0.8× as long as fifth tarsal segment.

*Metasoma.* First tergite 2.0× longer than its apical width, and apical width 3.4× its minimum width, with large dorsope basally (Fig. 8H); first tergite smooth basally, striate laterally (Fig. 8H); following tergites smooth and shiny; ovipositor sheath striate basally, 0.2× as long as fore wing; ovipositor long and straight (Fig. 7B).

*Colour.* Dark brown to black; face, basal part of antenna and of ovipositor sheath and ovipositor dark brown; wing membrane infuscate, pterostigma and veins dark brown.

#### Remarks.

This new species can be distinguished from related species by its unique “simple” antenna. Only S. (E.) sichuanensis Wang, 1986 shares this character with new species, but the new species differs from it as follows: (i) antenna with 24 antennomeres, scapus 7.5× longer than its maximum width (antenna with 21 antennomeres, scapus 5.7× longer than maximum width in *S. sichuanensis*); (ii) first metasomal tergite 2.0× longer than its apical width, striate laterally (first tergite 2.5× longer than its apical width, smooth laterally); (iii) propodeum without basal carina (propodeum with basal carina); (iv) ovipositor long and straight (ovipositor curved apically).

#### Biology.

Unknown.

#### Distribution.

Oriental: China (Yunnan).

#### Etymology.

Named after the laterally striate first tergite; “lateralis” is Latin for “of the side” and “stria” is Latin for “line”.

### Streblocera (Eutanycerus) lienhuachihensis

Taxon classificationAnimaliaHymenopteraBraconidae

Chou, 1990

5FAF1BC8-BFD5-515F-9D9F-CC75DA5EFB6A

Figures 9A–C

Streblocera (Cosmophoridia) lienhuachihensis Chou, 1990: 97.
Streblocera (Eutanycerus) lienhuachihensis ; Chen and van Achterberg, 1997: 115.

#### Material.

1♀, SE China, Fujian Province, Mt Wuyi, Huanggan, 22. vii.1986, Ming-hui Liu, average altitude 2000m; 1♀, same label data, but 26. ix.1981, Juchang Huang; 3♀, SE China, Fujian Province, Fuzhou city, Mt Wuyi, Tongmu, 23.vii.1988, jian-wen Chen, average altitude 1400m; 1♀, SE China, Fujian Province, Mt. Wuyi, Sangang, 12. x. 1986, Zhi-wu Xu, average altitude 900m; 1♀, SW China, Yunnan Province, Xishuangbanna city, Menglun, 17. ix. 1988, Juchang Huang, average altitude 680m; 1♀, SW China, Yunnan Province, Xishuangbanna city, Menglun, 16. ix. 1988, Jianquan Yang, average altitude 680m; 1♀, SW China, Yunnan Province, Xishuangbanna city, Menglun, 14. ix. 1988, Li-qin Zhang, average altitude 680m; 4♀, NE China, Jilin Province, Changchun city, Tuchengzi, Yulou, 22. viii. 2012, Yingying Zhao, average altitude 200m.

#### Distribution.

Oriental: China (Fujian, Taiwan and Yunnan) and Palaearctic: China (Hubei, Jilin).

**Figure 9.**
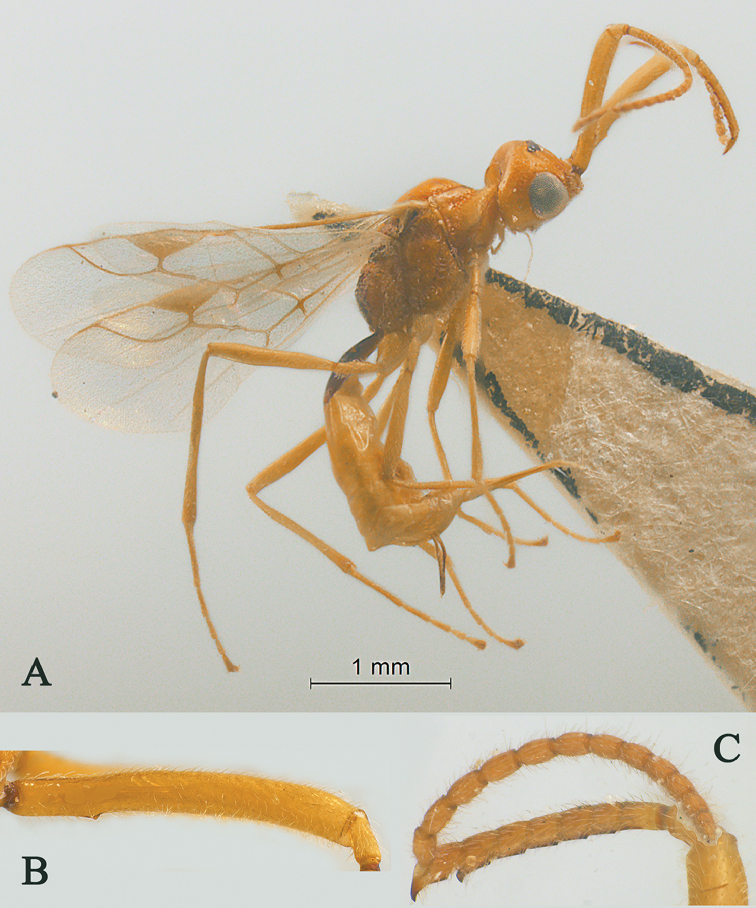
Streblocera (Eutanycerus) lienhuachihensis Chou, 1990, ♀ **A** habitus, lateral aspect **B** scapus **C** first to seventh flagellomeres.

### Streblocera (Eutanycerus) okadai

Taxon classificationAnimaliaHymenopteraBraconidae

Watanabe, 1942

0298174A-7B21-5951-89CE-53DBC166C22D

Figures 10A–C

Streblocera okadai Watanabe, 1942: 10; Maeto and Nagai 1985: 729.
Streblocera (Cosmophoridia) okadai ; Chou, 1990: 100; Chao 1993: 66.
Streblocera (Eutanycerus) okadai ; Chen and van Achterberg, 1997: 117; Belokobylskij 2000: 296.
Streblocera orientalis Chao, 1964: 154. Synonymised by Chao, 1993.
Streblocera zhongmouensis J. Wang, 1982: 61. Synonymised by Chao, 1993.
Streblocera shaanxiensis C. Wang, 1984: 411. Synonymised by Chao, 1993.
Streblocera flava You and Xiong, 1988: 167. Synonymised by Chao, 1993.

#### Material.

1♀, NW China, Qinghai Province, Xining city, Botanical garden, 21. vi. 2008, Qiong Zhao, average altitude 2300m; 1♀1♂, C China, Hunan Province, Zhangjiajie city (29°13N, 110°26E), 21. iv. 2015, Jun Li, average altitude 264m;1♀, C China, Shanxi Province, Changzhi city, Mt Taihang garden, 29. ix. 2010, Jun-li Yao, average altitude 1500m.

#### Biology.

Reared from *Medythia nigrobilineata* Motschulsky and *Medythia suturalis* Motschulsky (Chrysomelidae). This species is attracted to light (Yu et al. 2016).

#### Distribution.

Oriental: China (Fujian, Guizhou, Hunan, Jiangxi, Taiwan, Yunnan and Zhejiang) and Palaearctic: China (Anhui, Hebei, Henan, Hubei, Jiangsu, Jilin, Liaoning, Qinghai, Shaanxi, Shanxi, Shandong), Japan, Korea and Russia.

**Figure 10.**
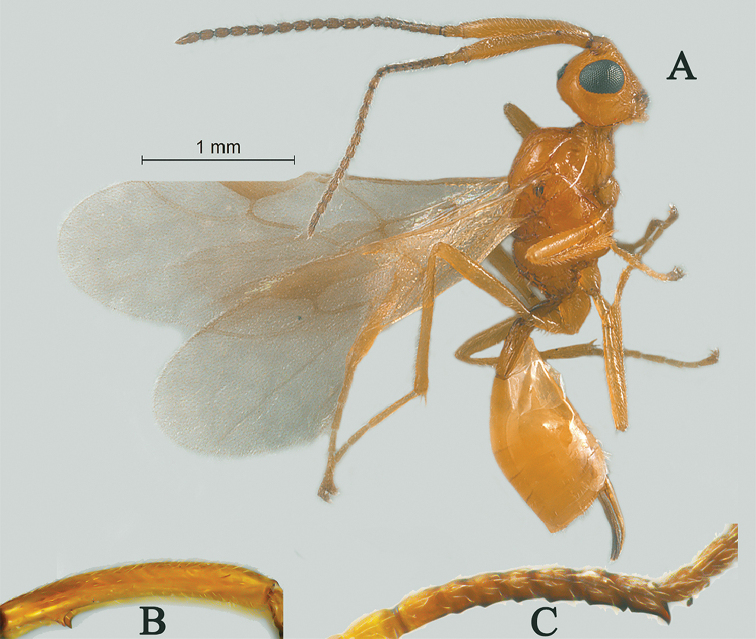
Streblocera (Eutanycerus) okadai Watanabe, 1942, ♀ **A** habitus, lateral aspect **B** scapus **C** first to seventh flagellomeres.

### Streblocera (Eutanycerus) opima

Taxon classificationAnimaliaHymenopteraBraconidae

Chou, 1990

B6833CCF-DB9C-58CB-99C8-19767BDE07E8

Figures 11A, B

Streblocera (Cosmophoridia) opima Chou, 1990: 101.
Streblocera (Eutanycerus) opima ; Chen and van Achterberg, 1997: 118.

#### Material.

1♀, SE China, Fujian Province, Mt Wuyi, Xingcun, 10. xi. 1987, Jia-hua Chen, average altitude 200m.

#### Biology.

Unknown.

#### Distribution.

Oriental: China (Fujian, Taiwan). New record for mainland China.

**Figure 11.**
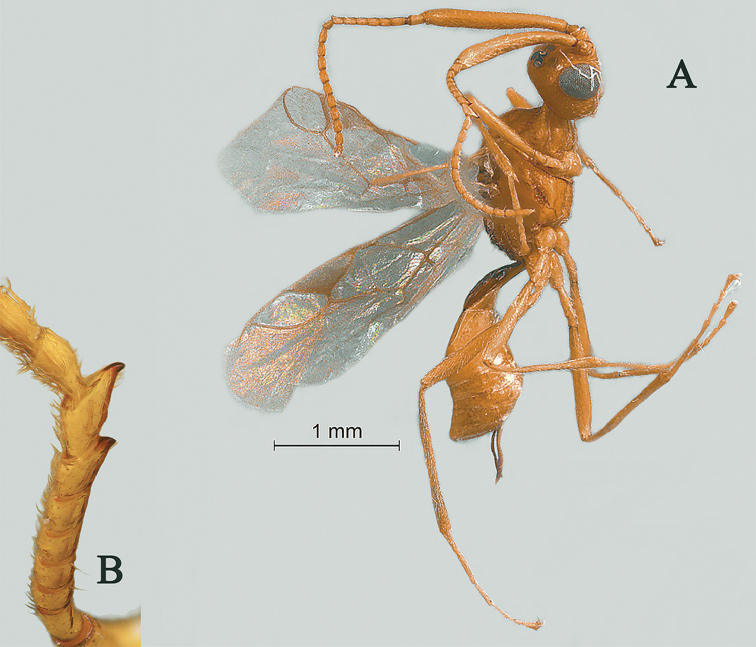
Streblocera (Eutanycerus) opima Chou, 1990, ♀ **A** habitus, lateral aspect **B** first to seventh flagellomeres.

### Streblocera (Eutanycerus) sungkangensis

Taxon classificationAnimaliaHymenopteraBraconidae

Chou, 1990.

09BFA7CB-1B77-5E69-A280-4DF7B5CF53AB

Figures 12A–C

Streblocera (Cosmophoridia) sungkangensis Chou, 1990:103.
Streblocera (Eutanycerus) sungkangensis ; Chen and van Achterberg, 1997: 119.

#### Type material.

1♀, NE China, Jilin Province, Mt. Changbai, Lushuihe, 29.vii.1989, Jian-quan Yang, average altitude 500m.

#### Biology.

Unknown.

#### Distribution.

Oriental (Guizhou, Taiwan, Zhejiang) and Palaearctic (Jilin). New record for Palaearctic region.

**Figure 12.**
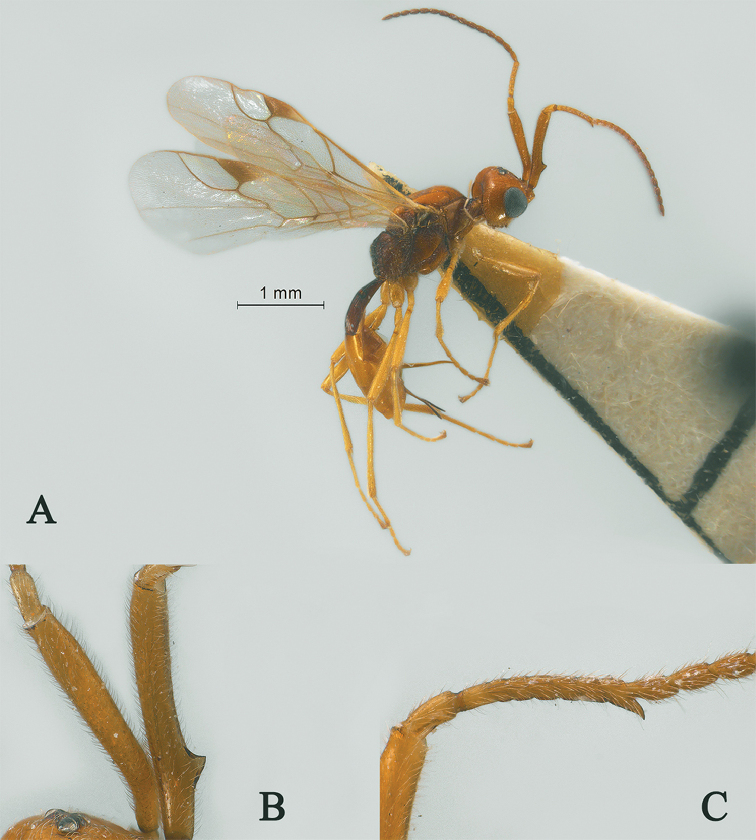
Streblocera (Eutanycerus) sungkangensis Chou, 1990, ♀ **A** habitus, lateral aspect **B** scapus **C** first to seventh flagellomeres.

### Streblocera (Eutanycerus) tsuifengensis

Taxon classificationAnimaliaHymenopteraBraconidae

Chou, 1990

8D513094-AB93-5CF4-8D19-5B6C4294A429

Figures 13A–C

Streblocera (Cosmophoridia) tsuifengensis Chou, 1990: 97.
Streblocera (Eutanycerus) tsuifengensis ; Chen and van Achterberg, 1997: 115.

#### Material.

2♀, SE China, Fujian Province, Mt. Meihua, 28. ix. 1988, Bao-bin Guan, average altitude 900m; 1♀, SE China, Fujian Province, Mt. Wuyi, Guadun, 28. vii. 1986, Hong Zhang, average altitude 1800m; 1♀, SE China, Fujian Province, Mt. Wuyi, Guading, 23. viii. 1986, Jianhua Ge, average altitude 1800m; 1♀, C China, Hubei Province, Shennongjia, Muyu, 6. viii. 1988, Jianquan Yang, average altitude 1200m; 1♀, C China, Hubei Province, Shennongjia, Muyu, 6. viii. 1988, Juchang Huang, average altitude 1200m.

#### Biology.

Unknown.

#### Distribution.

Oriental: China (Fujian, Taiwan) and Palaearctic: China (Hubei). New record for mainland China and new record for Palaearctic region

**Figure 13.**
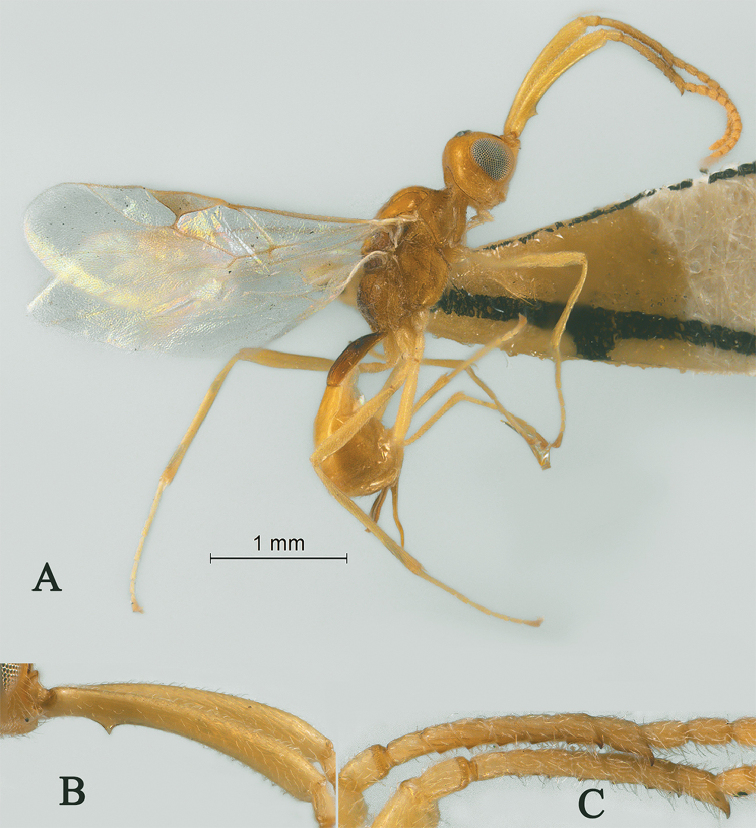
Streblocera (Eutanycerus) tsuifengensis Chou, 1990, ♀ **A** habitus, lateral aspect **B** scapus **C** first to seventh flagellomeres.

### Streblocera (Eutanycerus) uncifera

Taxon classificationAnimaliaHymenopteraBraconidae

Li, Chen & van Achterberg sp. nov.

25B64807-4AD4-5656-B523-D71DC8B322D4

http://zoobank.org/DDE2F698-0531-4710-B0C1-5162442C805E

**Figure 14.**
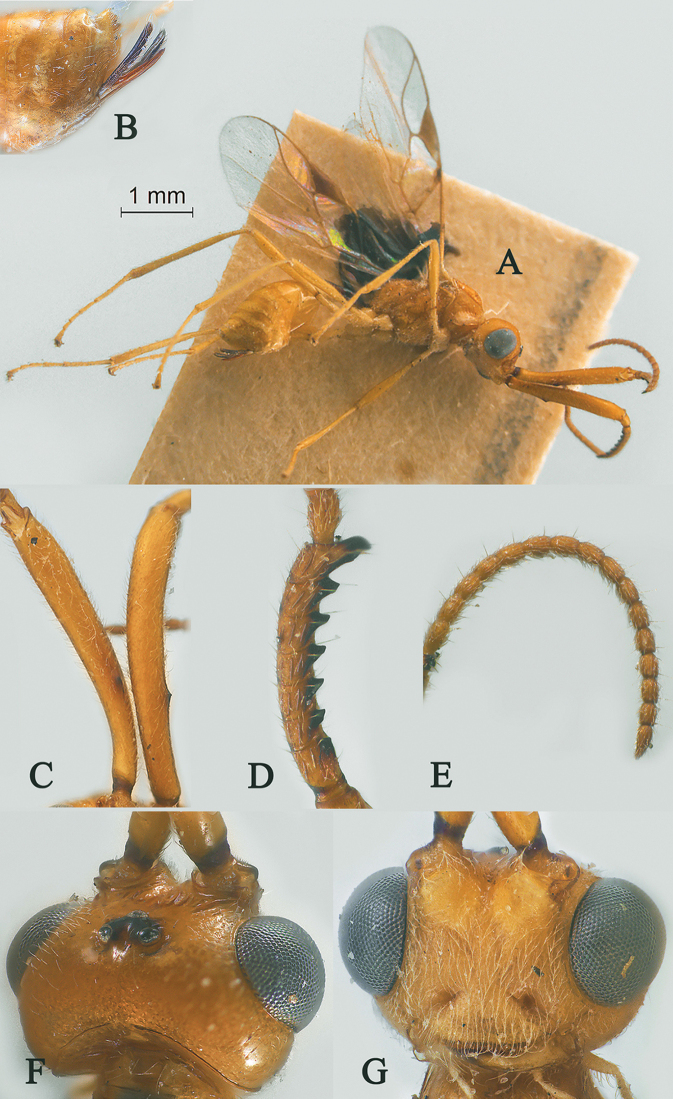
Streblocera (Eutanycerus) uncifera Li, Chen and van Achterberg, sp. nov., ♀ **A** abitus, lateral aspect **B** ovipositor and its sheaths, lateral aspect **C** scapus **D** first to seventh flagellomeres, lateral aspect **E** eighth to twenty-first flagellomeres, lateral aspect **F** head, dorsal aspect **G** head, anterior aspect.

**Figure 15.**
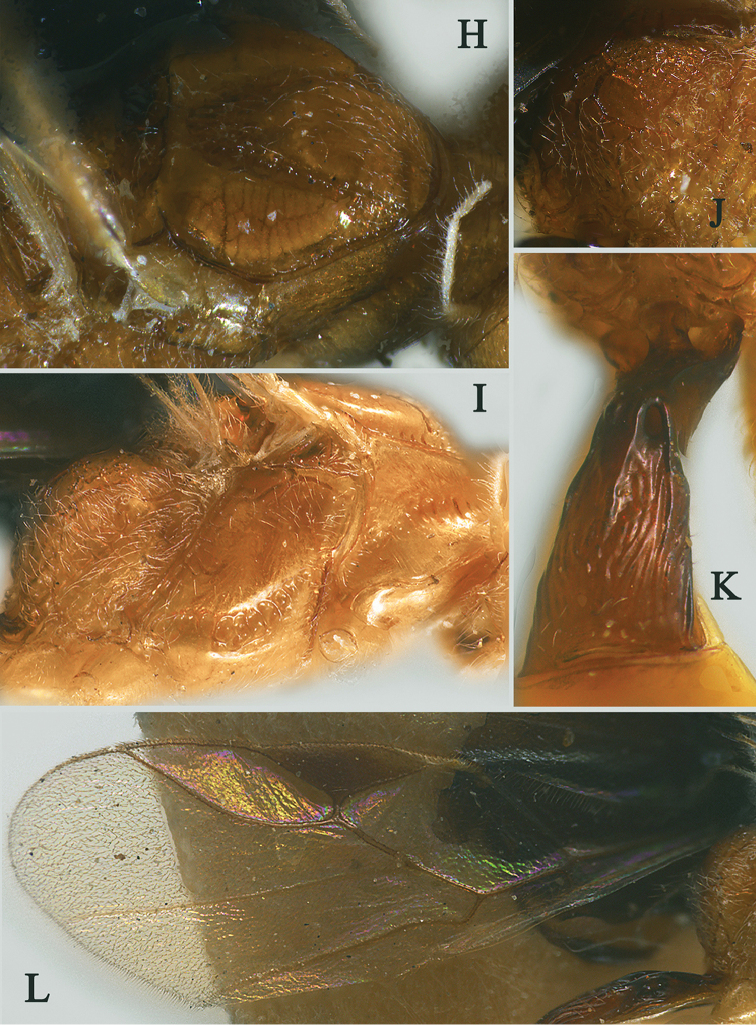
Streblocera (Eutanycerus) uncifera Li, Chen and van Achterberg, sp. nov., ♀ **H** mesosoma, dorsal aspect **I** mesosoma, lateral aspect **J** propodeum, dorsal aspect **K** first metasomal tergites, dorsal aspect **L** fore wing.

Figures 14A–G , 15H–L

#### Type material.

Holotype, ♀, SW China, Yunnan Province, Xishuangbanna, 14.ix.1988, Li-qin Zhang.

#### Description.

Holotype, ♀, length of antenna 3.1mm, of fore wing 3.5 mm, and of body 4.4 mm (Fig. 14A).

*Head.* Antenna with 23 antennomeres and 0.9× as long as fore wing, 0.8× as long as body (Fig. 14A); scapus long and slender, weakly expanded, 7.5× longer than its maximum width, evenly curved, with a small tooth-shaped horn, finely setose (Fig. 14C); first to seventh flagellomere modified: first to seventh flagellomeres serrate ventrally, serrated carina enlarged, respectively, and carina of seventh flagellomere with hook (Fig. 14D); first flagellomere 1.8× longer than second flagellomere, first, second and penultimate flagellomere 1.5, 0.8 and 1.4× as long as wide, respectively (Figs 14D, 14E); eye 1.2× longer than temple in dorsal view; temples slightly roundly narrowed behind eyes (Fig. 14F) ; ocelli small, OOL:OD:POL = 83:21:41 (Fig. 14F); frons and vertex largely punctate (Fig. 14F); occipital carina occipital carina nearly complete, interrupted medio-dorsally (Fig. 14F); face 1.6× wider than high, smooth (Fig. 14G); clypeus punctate, narrow than face, strongly convex, 2.5× wider than high (Fig. 14G); dorsal margin of clypeus slightly above level of ventral margin of eye anterior (Fig. 14G); tentorial pits large (Fig. 14G); malar suture shallow and narrow, length of malar space 1.4× basal width of mandible (Fig. 14G); mandibles long and slender, nearly completely overlapping when closed (Fig. 14G).

*Mesosoma.* Length of mesosoma 2.1× its height (Fig. 15I); side of pronotum crenulated anteriorly and medially, largely smooth and shiny (Fig. 15I); propleuron smooth and shiny (Fig. 15I); mesopleuron smooth (Fig. 15I); prepectal medio-ventral carina present (Fig. 15I); episternal scrobe short and wide (Fig. 15I); precoxal sulcus long, wide and crenulate (Fig. 15I); mesonotum moderately sparsely setose, flat, smooth and, rugose anteriorly (Fig. 15H); notauli narrow, posteriorly rugose; mesoscutum sparsely setose, flattened (Fig. 15H); scutellar sulcus wide and smooth with one distinct crenula (Fig. 15H); scutellum flat, smooth (Fig. 15H); metapleuron reticulate (Fig. 15I); propodeum with rather short basal carina and pentagon-shaped median area dorsally, laterally rugulose (Fig. 15J).

*Wings.* Fore wing (Fig. 15L): vein 1-SR+M absent; vein 1-R1 0.8× as long as pterostigma; vein SR1+3-SR curved; r:2-SR = 11:53; vein r issued from middle of pterostigma; vein m-cu cross vein 2-SR; vein cu-a nearly as long as vein 1-CU1 and postfurcal.

*Legs.* Fore leg: tibia 5.4× longer than coxa, 1.2× longer than femur; middle leg: tibia 3.7× longer than coxa, 1.1× longer than femur; hind leg: tibia 4.4× longer than coxa, 1.4× longer than femur; hind coxa smooth, 1.2× longer than wide; femur, tibia and basitarsus 8.0, 13.6 and 8.9× longer than wide, respectively; hind basitarsus 0.4× as long as hind tibia, and 0.8× as long as combined second to fifth tarsal segments; hind fourth tarsal segment 0.9× as long as fifth tarsal segment.

*Metasoma.* First tergite robust, 1.7× longer than its apical width, apical width 3.4× longer than its minimum width, with dorsope basally but no laterope (Fig. 15K); first tergite smooth basally, rugose laterally (Fig. 15K); following tergites smooth and shiny; ovipositor sheath robust and base half crenulate, 0.1× as long as fore wing; ovipositor robust curved upwards (Fig. 15B).

*Colour.* Yellowish brown to brown; palpi pale yellow; legs yellowish brown; face, antenna, scutellum, metanotum brown; wing membrane hyaline, pterostigma and veins brown; ovipositor sheath and ovipositor dark brown.

#### Remarks.

This new species is similar to S. (E.) hsiufui You, 1999, but differs from it as follows: (i) first to seventh flagellomeres serrate ventrally, only the carina of seventh flagellomere with hook (first to seventh flagellomeres serrate ventrally and all with hook in *S. hsiufui*); (ii) first metasomal segment more robust, 1.7× longer than its apical width (first metasomal segment 2.3× longer than its apical width); (iii) ovipositor sheath more robust and ovipositor curved upwards (ovipositor sheath slender and ovipositor wave-like bent); (iv) body yellowish brown to brown (body dark brown).

#### Biology.

Unknown.

#### Distribution.

Oriental: China (Yunnan).

#### Etymology.

Named after hook bearing seventh flagellomere: “uncus” is “hook” in Latin and “fero” is Latin for “carry”.

### Streblocera (Streblocera) emarginata

Taxon classificationAnimaliaHymenopteraBraconidae

Chou, 1990

4E6E2282-63B7-566D-98EF-702DF668CB21

Figure 16

Streblocera (Streblocera) emarginata Chou, 1990: 107; Chen and van Achterberg 1997: 121.

#### Material.

1♀, C China, Hubei Province, Shennongjia, Muyu, 5.viii.1988, Li-qin Zhang, average altitude 1200m.

#### Biology.

Unknown.

#### Distribution.

Oriental: China (Taiwan) and Palaearctic: (Hubei). New record for mainland China.

**Figure 16.**
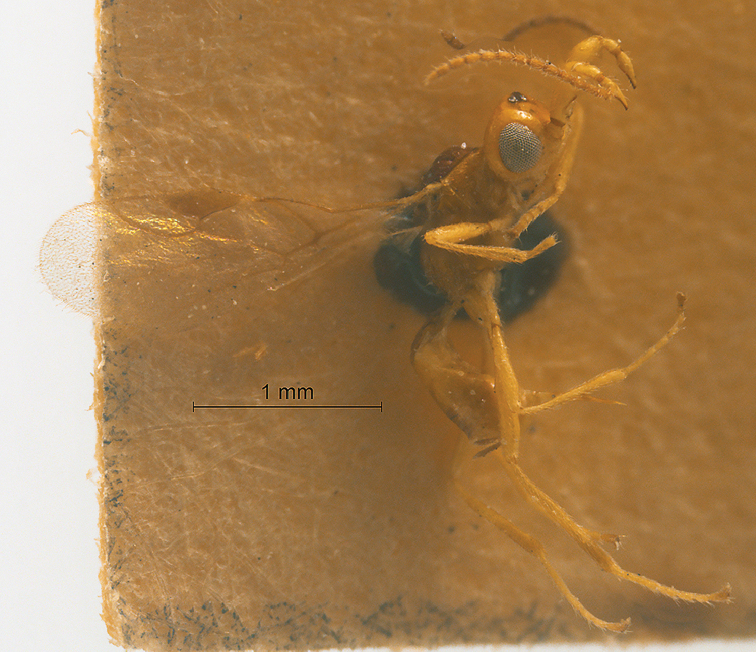
Streblocera (Streblocera) emarginata Chou, 1990, ♀, habitus, lateral aspcet.

### Streblocera (Streblocera) interrupta

Taxon classificationAnimaliaHymenopteraBraconidae

Li, Chen & van Achterberg sp. nov.

F102C7BE-F2BB-58B3-A90E-FA28D6337AEE

http://zoobank.org/044A2CC2-402A-4396-A92F-20893FD62B2E

**Figure 17.**
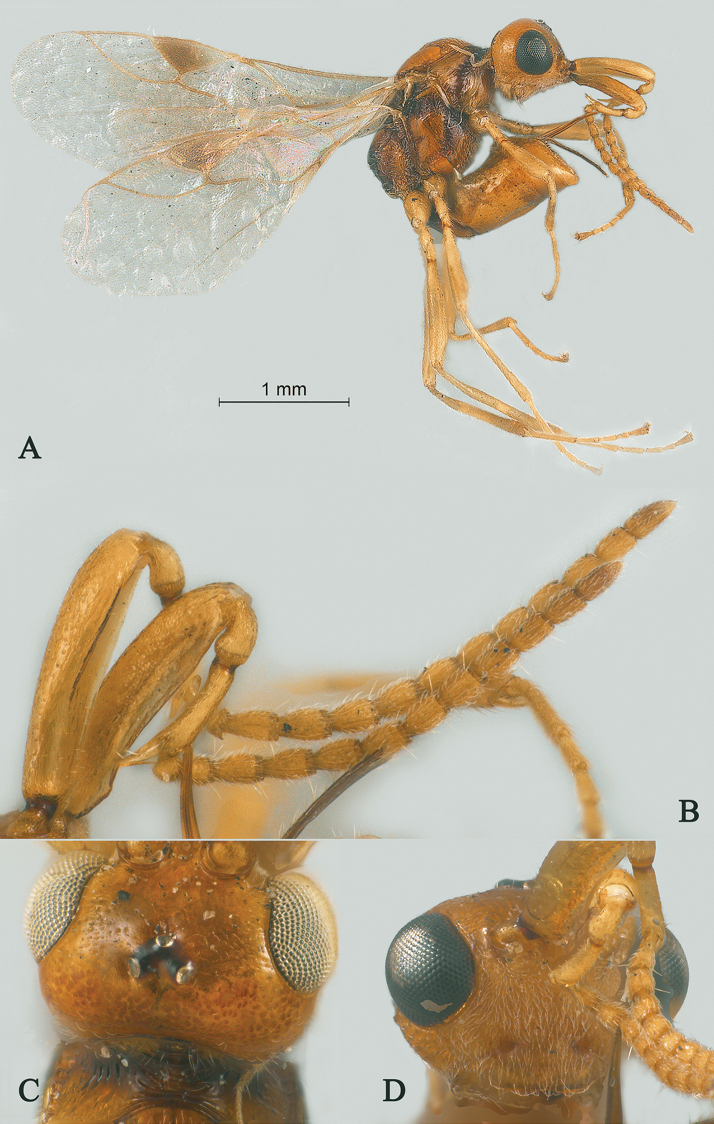
Streblocera (Streblocera) interrupta Li, Chen and van Achterberg, sp. nov., ♀ **A** habitus, lateral aspect **B** antenna **C** head, dorsal aspect **D** head, anterior aspect.

**Figure 18.**
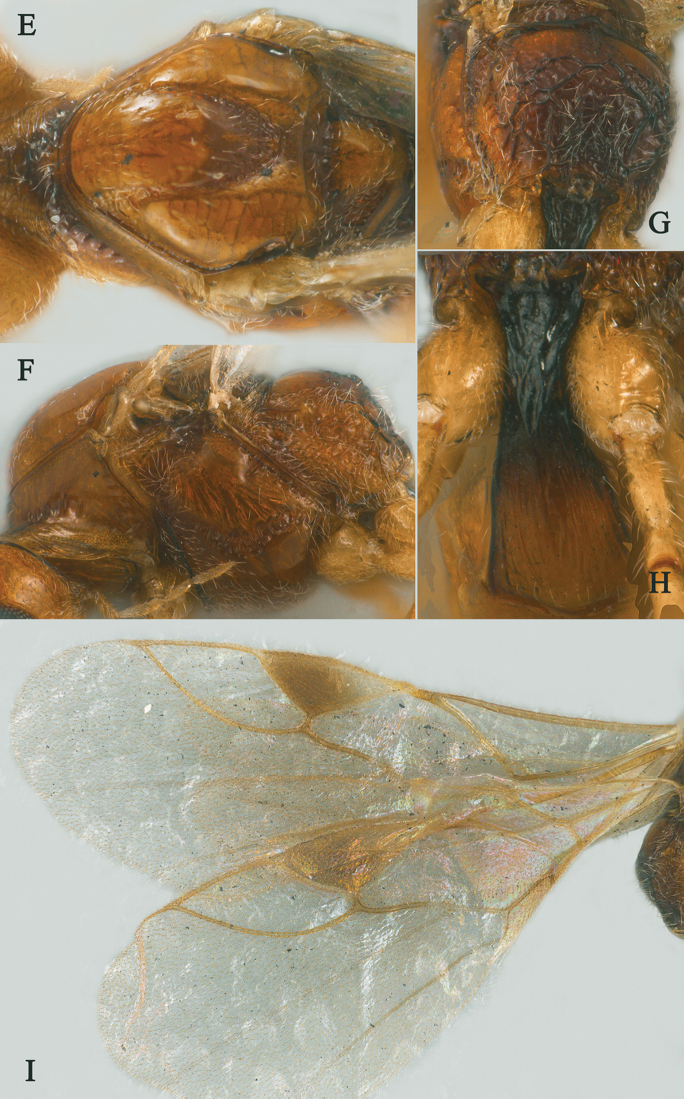
Streblocera (Streblocera) interrupta Li, Chen and van Achterberg, sp. nov., ♀ **E** mesosoma, dorsal aspect **F** mesosoma, lateral aspect **G** propodeum, dorsal aspect **H** first metasomal tergites, dorsal aspect **I** fore wing.

Figures 17A–D , 18E–I

#### Type material.

Holotype, ♀, C China, Hubei Province, Shennongjia, Honghua, 21.viii.2000, Qing-E Ji, average altitude 1800m. Paratype: 1♀, same label data as holotype.

#### Description.

Holotype, ♀, length of antenna 2.2 mm, of fore wing longer than 3.1 mm, and of body 3.3 mm.

*Head.* Antenna with 15 antennomeres and 0.7× as long as body (Fig. 17A); scapus rather stout and straight, weakly expanded, 4.4× longer than its maximum width, with distinct carina ventrally which is curved from middle, sparsely setose (Fig. 17B); antenna geniculated at first flagellomere and first flagellomere modified: first flagellomere slender and subcylindrical with long hook apically, second flagellomere inserted on first flagellomere near its middle (Fig. 17B); first flagellomere 3.0× longer than second flagellomere, first, second and penultimate flagellomere 7.4, 2.1 and 1.9× longer than wide, respectively (Fig. 17B); eye 1.8× longer than temple in dorsal view, inner side of eye curved (Fig. 17C); temples roundly narrowed behind eyes; ocelli small, OOL:OD:POL = 22:4:9 (Fig. 17C); frons and vertex smooth to punctate (Fig. 17C); occipital carina nearly complete, very narrowly interrupted and straight dorsally (Fig. 17C); face 1.7× wider than high, smooth and densely setose (Fig. 17D); clypeus smooth, narrow than face, slightly convex, 3.5× wider than high (Fig. 17D); dorsal margin of clypeus far below level of ventral margin of eye in anterior view (Fig. 17D); tentorial pits large (Fig. 17D); malar suture wide, length of malar space 1.8× basal width of mandible (Fig. 17D).

*Mesosoma.* Length of mesosoma 1.4× its height (Fig. 18F); side of pronotum crenulated anteriorly, largely smooth and shiny (Fig. 18F); propleuron smooth (Fig. 18F); mesopleuron smooth and shiny (Fig. 18F); prepectal medio-ventral carina present (Fig. 18F); episternal scrobe short (Fig. 18F); precoxal sulcus long, narrow and crenulate (Fig. 18F); mesonotum sparsely setose, convex, smooth and slightly shiny (Fig. 18E); notauli narrow and crenulated to rugose; mesoscutum sparsely setose, flat (Fig. 18E); scutellar sulcus smooth with one distinct crenula (Fig. 18E); scutellum convex, smooth (Fig. 18E); metapleuron largely reticulate (Fig. 18F); propodeum reticulate laterally rugose (Fig. 18G).

*Wings.* Fore wing (Fig. 18I): vein 1-SR+M absent; vein 1-R1 0.8× as long as pterostigma; vein SR1+3-SR curved; r:2-SR = 5:27; vein r issued from middle of pterostigma; vein m-cu cross vein 2-SR; vein cu-a longer than vein 1-CU1, postfurcal.

*Legs.* Fore leg: tibia 2.7× longer than coxa, 0.9× as long as femur, and femur flat, 3.0× longer than wide; middle leg: tibia 6.2× longer than coxa, 1.3× longer than femur; hind leg: tibia 4.0× longer than coxa, 1.4× longer than femur; hind coxa smooth, 1.2× longer than wide; hind femur, tibia and basitarsus 6.4, 13.3 and 8.8× longer than wide, respectively; hind basitarsus 0.3× as long as tibia, and 0.5× as long as combined second to fifth tarsal segments; hind fourth tarsal segment 0.8× as long as fifth tarsal segment.

*Metasoma.* First tergite 1.9× longer than its apical width, apical width 2.6× its minimum width, with dorsope at basal 0.3 (Fig. 18H); first tergite basally rugulose, laterally striate to rugulose (Fig. 18H); following tergites smooth and shiny; ovipositor sheath and ovipositor typical, ovipositor curved to pointing upward apically (Fig. 17A).

*Colour.* Yellowish brown to dark brown; palpi, basal antenna and legs yellowish brown; antenna and head largely brown; wing membrane hyaline, pterostigma and veins brown.

#### Remarks.

This new species is similar to S. (S.) latiscapus Belokobylskij, 2000, but differs from it as follows: (i) antenna with 15 antennomeres, first flagellomere 5.8× longer than second flagellomere (antenna with 17–18 antennomeres, first flagellomere 2.8–3.0× longer than second flagellomere in *S. latiscapus*); (ii) eye 1.8× longer than temple in dorsal view, inner side of eye curved (eye 1.6× longer than temple in dorsal view and inner side of eye straight).

#### Biology.

Unknown.

#### Distribution.

Palaearctic: China (Hubei).

#### Etymology.

Named after the narrowly interrupted occipital carina; “interruptus” is Latin for “broken apart”.

### Streblocera (Streblocera) jezoensis

Taxon classificationAnimaliaHymenopteraBraconidae

Belokobylskij, 2000

C8E80496-7827-507E-B79B-63102621CD83

Figure 19

Streblocera (Streblocera) jezoensis Belokobylskij, 2000: 282.

#### Material.

1♀, SE China, Fujian Province, Nanping city, Guangze, Daqing, 1. viii. 2002, Cunzhu Dong, average altitude 380m; 1♀, SE China, Fujian Province, Nanping city, Guangze, Qikeng, 30. vii. 2002, Jianquan Yang, average altitude 300m

#### Biology.

Unknown.

#### Distribution.

Oriental: China (Fujian) and Palaearctic: Japan. New record for China and for Oriental region.

**Figure 19.**
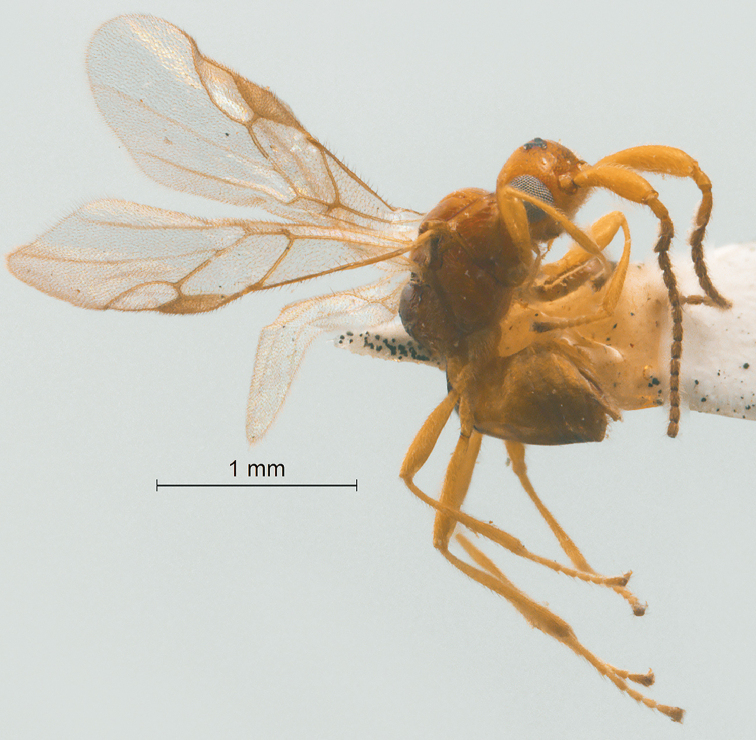
Streblocera (Streblocera) jezoensis Belokobylskij, 2000, ♀, habitus, lateral aspcet.

### Streblocera (Streblocera) spasskensis

Taxon classificationAnimaliaHymenopteraBraconidae

Belokobylskij, 2000

51C5F27D-A772-5A3E-8C49-FA6E85881A20

Figures 20A, B

Streblocera (Streblocera) spasskensis Belokobylskij, 2000: 290, 313; Lee et al. 2016: 471.

#### Material.

1♀, C China, Hubei Province, Shennongjia, 18. viii. 1988, Juchang Huang.

#### Biology.

Unknown.

#### Distribution.

Palaearctic: China (Hubei), Korea, Russia. New record for China.

**Figure 20.**
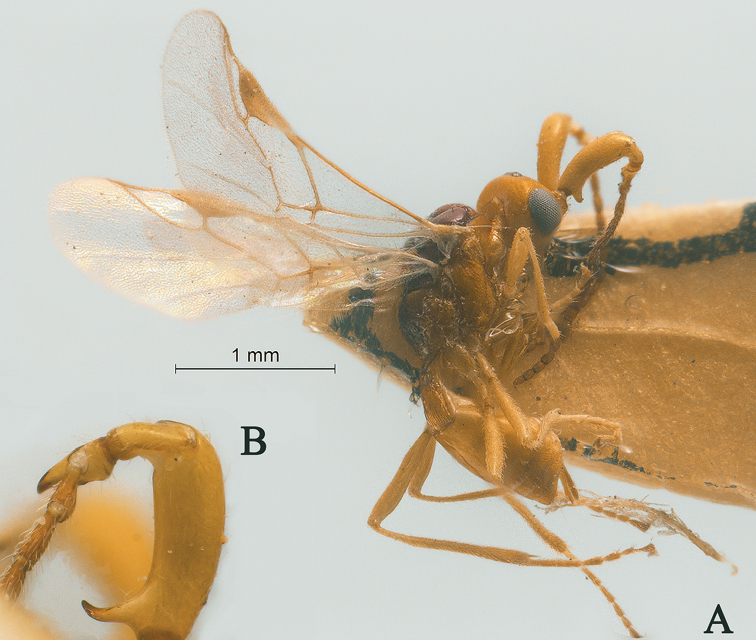
Streblocera (Streblocera) spasskensis Belokobylskij, 2000, ♀ **A** habitus, lateral aspcet **B** scapus.

### Streblocera (Streblocera) stigenbergae

Taxon classificationAnimaliaHymenopteraBraconidae

Li, Chen & van Achterberg sp. nov.

0539C586-4DA2-5690-BCFB-EA61956528F7

http://zoobank.org/9DE58198-A9BD-4097-850A-817E3831A437

**Figure 21.**
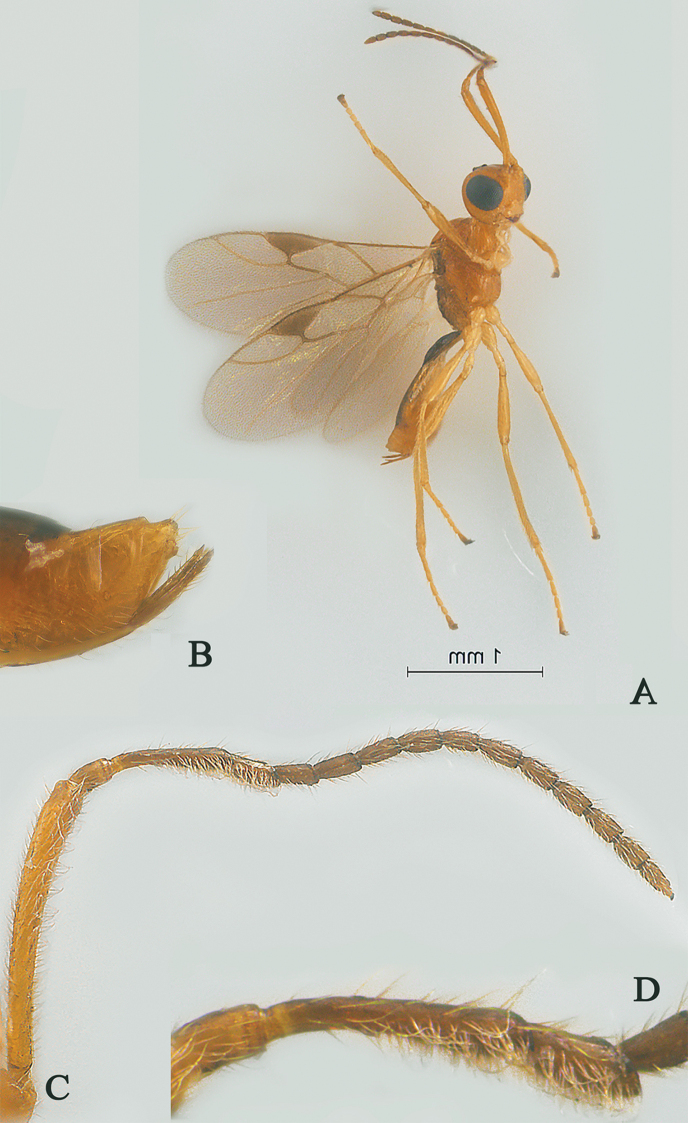
Streblocera (Streblocera) stigenbergae Li, Chen and van Achterberg, sp. nov., ♀ **A** habitus, lateral aspect **B** ovipositor and its sheaths, lateral aspect **C** antenna **D** first to second flagellomeres, lateral aspect.

**Figure 22.**
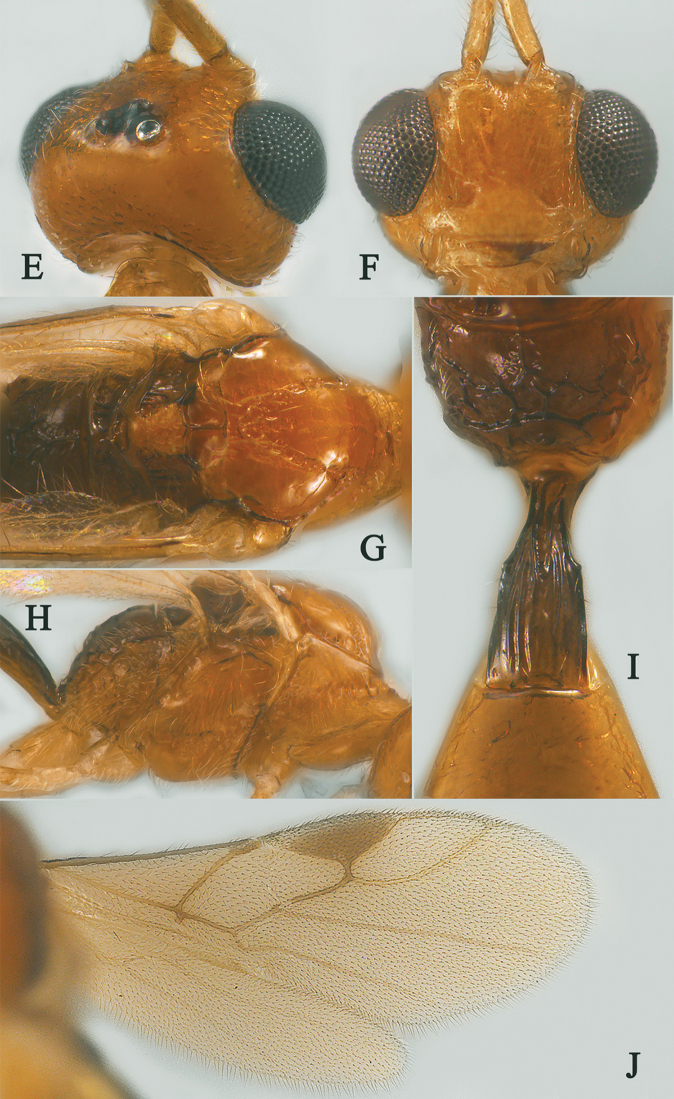
Streblocera (Streblocera) stigenbergae Li, Chen and van Achterberg, sp. nov., ♀ **E** head, dorsal aspect **F** head, anterior aspect **G** mesosoma, dorsal aspect **H** mesosoma, lateral aspect **I** propodeum and first metasomal tergites, dorsal aspect **J** wings.

Figures 21A–D , 22E–J

#### Type material.

Holotype, ♀, SW China, Yunan Province, Wenshan City, Malipo County, Zhongzaichapai, 22.vi. 2017, Yan-Qiong Peng, 1972m.

#### Description.

Holotype, ♀, length of antenna 1.8mm, of fore wing 2.0 mm, and of body 2.2 mm.

*Head.* Antenna with 15 antennomeres and 0.9× as long as fore wing, 0.8× as long as body (Fig. 21A); scapus long and slender, weakly expanded, 5.2× longer than its maximum width, evenly curved, without horn, finely setose (Fig. 21C); first and second flagellomeres densely setose and modified: first flagellomere slender and long, second flagellomere stout and weakly expanded (Fig. 21D); first flagellomere 2.6× longer than second flagellomere, first, second and penultimate flagellomere 5.1, 1.9 and 1.9× longer than wide, respectively (Fig. 21C); eye 1.8× longer than temple in dorsal view; temples roundly narrowed behind eyes (Fig. 22E); ocelli medium-sized, OOL:OD:POL = 41:14:23 (Fig. 22E); frons and vertex largely smooth (Fig. 22E); occipital carina nearly complete, narrowly interrupted medio-dorsally (Fig. 22E); face nearly 1.5× wider than high, smooth and shiny (Fig. 22F); clypeus smooth and shiny, wider than face, strongly convex, 3.0× wider than high (Fig. 22F); dorsal margin of clypeus above level of ventral margin of eye anterior (Fig. 22F); tentorial pits large (Fig. 22F); malar suture short, length of malar space 0.5× basal width of mandible (Fig. 22F); mandibles long, nearly completely overlapping when closed (Fig. 22F).

*Mesosoma.* Length of mesosoma 2.3× its height (Fig. 22H); side of pronotum crenulated anteriorly and medially, largely smooth (Fig. 22H); propleuron smooth (Fig. 22H); mesopleuron smooth (Fig. 22H); prepectal medio-ventral carina present (Fig. 22H); episternal scrobe short and wide (Fig. 22H); precoxal sulcus wide and crenulate (Fig. 22H); mesonotum moderately sparsely setose, flat, smooth (Fig. 22G); notauli narrow, smooth, mesoscutum sparsely setose, flattened (Fig. 22G); scutellar sulcus very wide and smooth with one distinct crenula (Fig. 22G); scutellum flat, smooth (Fig. 22G); metapleuron reticulate (Fig. 22H); propodeum with rather long basal carina and pentagon-shaped median area, largely smooth (Fig. 22I).

*Wings.* Fore wing (Fig. 22J): vein 1-SR+M absent; vein 1-R1 0.4× as long as pterostigma; vein SR1+3-SR strongly curved; r:2-SR = 11:46; vein r issued behind middle of pterostigma; vein m-cu cross vein 2-SR; vein cu-a longer than vein 1-CU1 and postfurcal; basal and subbasal cells of fore wing similarly setose as other cells.

*Legs.* Fore leg: tibia 5.2× longer than coxa, 1.1× longer than femur; middle leg: tibia 3.9× longer than coxa, 1.1× longer than femur; hind leg: tibia 3.7× longer than coxa, 1.6× longer than femur; hind coxa smooth, 1.2× longer than wide; femur, tibia and basitarsus 5.6, 14.5 and 7.7× longer than wide, respectively; hind basitarsus 0.3× as long as hind tibia, and 0.6× as long as combined second to fifth tarsal segments; fourth hind tarsal segment 0.9× fifth tarsal segment.

*Metasoma.* First tergite slightly narrowed behind spiracle, 2.2× longer than its maximum width, apical width 2.9× its minimum width, without dorsope and laterope (Fig. 22I); first tergite rugose basally, striate laterally and subapically (Fig. 22I); following tergites smooth and shiny; ovipositor sheath 0.1× as long as fore wing; ovipositor strongly curved upwards (Fig. 21B).

*Colour.* Yellowish brown to dark brown; face, basal part of antenna, palpi and legs yellowish brown; scutellum, metanotum, brown; wing membrane hyaline, pterostigma and veins dark brown; second to thirteenth flagellomeres, propodeum and metasomal segments dark brown.

#### Remarks.

This new species can be distinguished from related species by the combination of 15 antennomeres, scapus without horn, first and second flagellomere densely setose and modified, occipital carina narrowly interrupted medio-dorsally, very wide scutellar sulcus and ovipositor short and strongly curved upwards.

#### Biology.

Unknown.

#### Distribution.

Oriental: China (Yunnan).

#### Etymology.

Named after the Swedish entomologist Dr Julia Stigenberg (Stockholm) for her contribution to the taxonomy of Euphorinae and for her help to the first author.

### Streblocera (Streblocera) tayulingensis

Taxon classificationAnimaliaHymenopteraBraconidae

Chou, 1990

25634BCA-4E89-58C8-A1FE-B62D62D26638

Figures 23A, B

Streblocera (Streblocera) tayulingensis Chou, 1990: 113; Chen and van Achterberg 1997: 123.

#### Material.

2♀, SE China, Fujian Province, Mt Wuyi, Huanggan, vii.1986, Jia-hua Chen, average altitude 2000m; 1♀, SE China, Fujian Province, Mt Wuyi, Sangang, 13.x.1980, Jia-hua Chen, average altitude 900m; 1♀, SE China, Fujian Province, Mt Wuyi, Tongmu, 5.viii.1998, Jianquan Yang, average altitude 1100m; 1♀, C China, Hubei Province, Shennongjia, Muyu, 7. viii. 1988, Juchang Huang, average altitude 1200m.1♀, C China, Hubei Province, Shennongjia, 18. viii. 1988, Juchang Huang.

#### Biology.

Unknown.

#### Distribution.

Oriental: China (Fujian, Taiwan, Zhejiang) and Palaearctic: China (Hubei).

#### Remarks.

The studied specimens differ as follows: scapus fairly long and weakly expanded, 5.0× longer than its maximum width (scapus more robust, expanded, 4.4–4.5× longer than its maximum width according to the original description).

**Figure 23.**
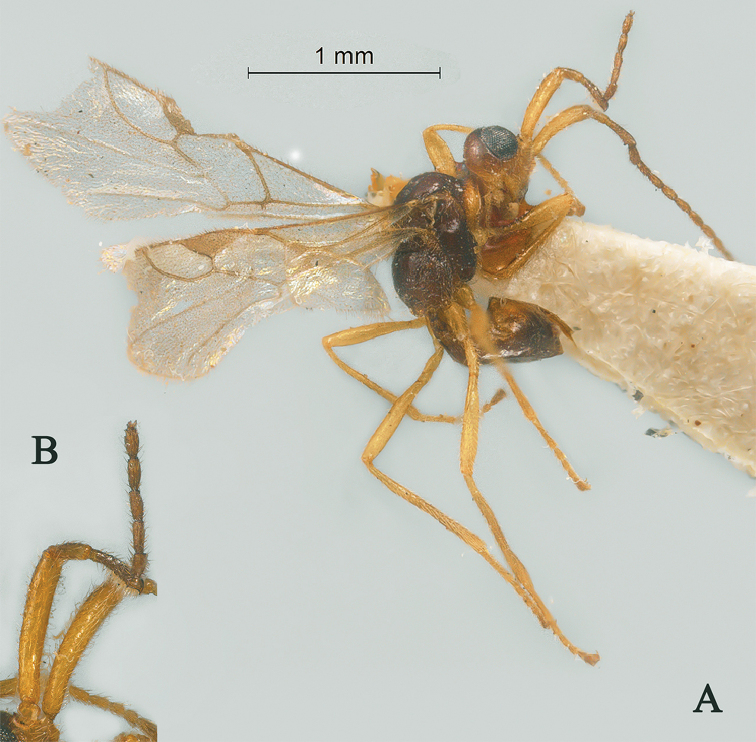
Streblocera (Streblocera) tayulingensis Chou, 1990, ♀ **A** habitus, lateral aspect **B** antenna.

### Streblocera (Streblocera) trullifera

Taxon classificationAnimaliaHymenopteraBraconidae

Li, Chen & van Achterberg sp. nov.

EACE5A4A-572B-544E-B9B6-032A56F3D250

http://zoobank.org/8C607D72-DA27-484E-BBC0-2C0B0157E654

**Figure 24.**
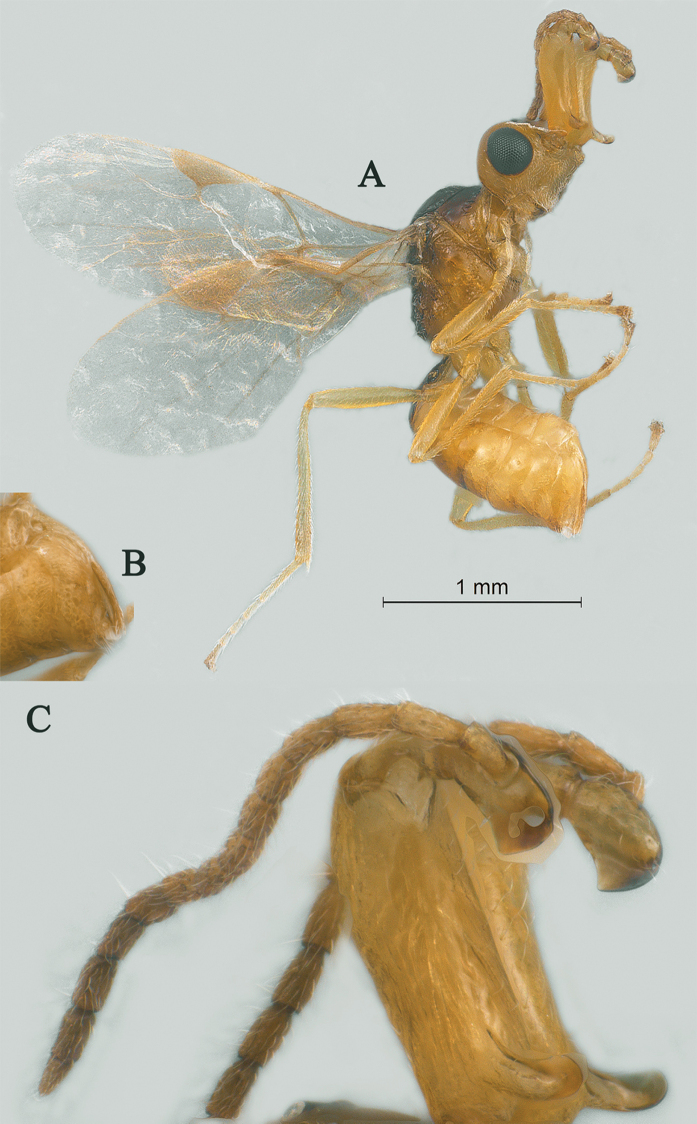
Streblocera (Streblocera) trullifera Li, Chen and van Achterberg, sp. nov., ♀ **A** habitus, lateral aspect **B** ovipositor and its sheath, lateral aspect **C** antenna.

**Figure 25.**
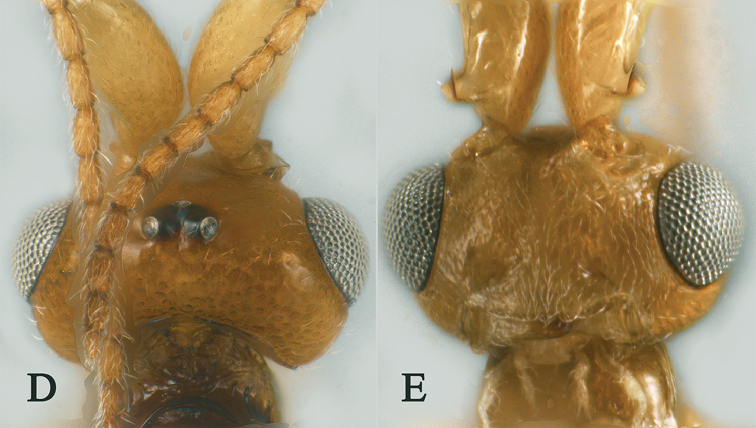
Streblocera (Streblocera) trullifera Li, Chen and van Achterberg, sp. nov., ♀ **D** head, dorsal aspect **E** head, anterior aspect.

**Figure 26.**
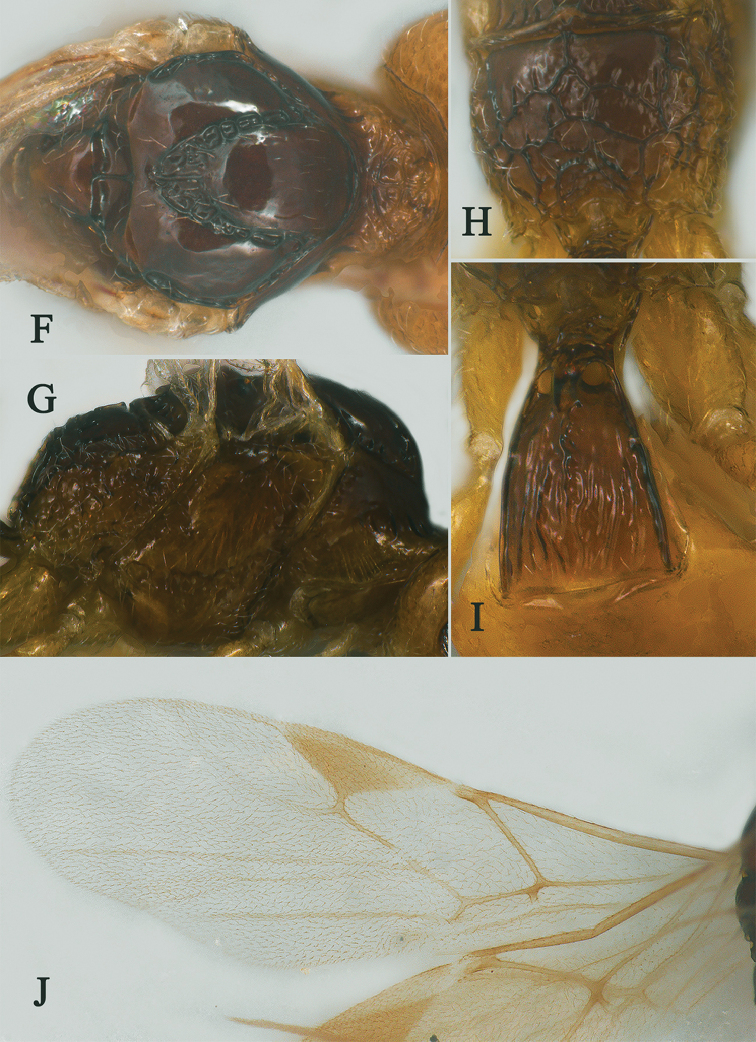
Streblocera (Streblocera) trullifera Li, Chen and van Achterberg, sp. nov., ♀ **F** mesosoma, dorsal aspect **G** mesosoma, lateral aspect **H** propodeum, dorsal aspect **I** first metasomal tergites, dorsal aspect **J** fore wing.

Figures 24A–C , 25D, E , 26F–J

#### Type material.

Holotype, ♀, NE China, Liaoning Province, Fuxin city, Sanba park, 27.vii.2012, Yingying Zhao, average altitude 200m.

#### Description.

Holotype, ♀, length of antenna 1.5 mm, of fore wing longer than 2.2 mm, and of body 2.5 mm.

*Head.* Antenna with 17 antennomeres and 0.6× as long as body (Fig. 24A); scapus stout, weakly expanded, 3.0× longer than its maximum width, with spoon-shaped horn basally, sparsely setose (Figs 24C, 25E); antenna geniculated at first flagellomere and first flagellomere modified: first flagellomere flat and rectangular with small hook, second flagellomere inserted on first flagellomere near its middle (Fig. 24C); first flagellomere 2.0× longer than second flagellomere, first, second and penultimate flagellomere 1.6, 2.0 and 2.5× longer than wide, respectively (Fig. 24C); eye 1.6× longer than temple in dorsal view (Fig. 19 D); temples roundly narrowed behind eyes (Fig. 25D); ocelli small, OOL:OD:POL = 47:9:21 (Fig. 25D); frons and vertex smooth (Fig. 25D); occipital carina nearly complete, narrowly interrupted medio-dorsally (Fig. 25D); face 2.2× wider than high, smooth to punctate (Fig. 25E); clypeus smooth, narrow than face, slightly convex, 3.1× wider than high (Fig. 25E); dorsal margin of clypeus above level of ventral margin of eye anterior (Fig. 25E); tentorial pits large (Fig. 25E); malar suture narrow, length of malar space 1.2× basal width of mandible (Fig. 25E); mandibles stout (Fig. 25E).

*Mesosoma.* Length of mesosoma 2.1× its height (Fig. 26G); side of pronotum crenulated anteriorly, largely striate (Fig. 26G); propleuron smooth (Fig. 26G); mesopleuron smooth (Fig. 26G); prepectal carina complete present (Fig. 26G); episternal scrobe short (Fig. 26G); precoxal sulcus long, narrow and crenulate (Fig. 26G); mesonotum sparsely setose, convex, smooth and finely shiny (Fig. 26F); notauli narrow and crenulated; mesoscutum sparsely setose, finely convex (Fig. 26F); scutellar sulcus wide and smooth with one distinct crenula (Fig. 26F); scutellum convex, smooth (Fig. 26F); metapleuron largely reticulate (Fig. 26G); propodeum with short basal carina and median area dorsally, laterally reticulate and partly smooth (Fig. 26H).

*Wings.* Fore wing (Fig. 26J): vein 1-SR+M absent; vein 1-R1 0.6× as long as pterostigma; vein SR1+3-SR largely unsclerotized and curved; r:2-SR = 9:51; vein r issued behind middle of pterostigma; vein m-cu cross vein 2-SR; vein cu-a longer than vein 1-CU1, postfurcal and finely stout.

*Legs.* Fore leg: tibia 4.2× longer than coxa, 1.3× longer than femur; middle leg: tibia 3.4× longer than coxa, 1.4× longer than femur; hind leg: tibia 4.0× longer than coxa, 1.4× longer than femur; hind coxa smooth, 1.4× longer than wide; hind femur, tibia and basitarsus 5.6, 10.6 and 5.2× longer than wide, respectively; hind basitarsus 0.3× as long as tibia, and 0.6× as long as combined second to fifth tarsal segments; hind fourth tarsal segment 0.7× as long as fifth tarsal segment.

*Metasoma.* First tergite 1.8× longer than its apical width, apical width 2.3× its minimum width, with large dorsope basally (Fig. 26I); first tergite basally rugulose, laterally striate to rugulose (Fig. 26I); following tergites smooth and shiny; ovipositor sheath and ovipositor short (Fig. 24B).

*Colour.* Yellowish brown to dark brown; palpi pale yellow; basal part of antenna, head and legs yellowish brown; antenna and mesopleuron largely brown; wing membrane hyaline, pterostigma and veins brown.

#### Remarks.

This new species can be distinguished from related species by its combination of 17 antennomeres, scapus with a spoon-shaped horn basally and vein SR1+3-SR of fore wing largely unsclerotized.

#### Biology.

Unknown.

#### Distribution.

Palaearctic: China (Liaoning).

#### Etymology.

Named after its spoon-shaped horn of the scapus: “trulla” is “little spoon” in Latin and “fero” is Latin for “carry”.

### Streblocera (Streblocera) zoroi

Taxon classificationAnimaliaHymenopteraBraconidae

Li, Chen & van Achterberg sp. nov.

F9749E00-0BE2-56DC-866E-1FC7140C005E

http://zoobank.org/31837179-9C29-48C5-AF37-0A3855202218

**Figure 27.**
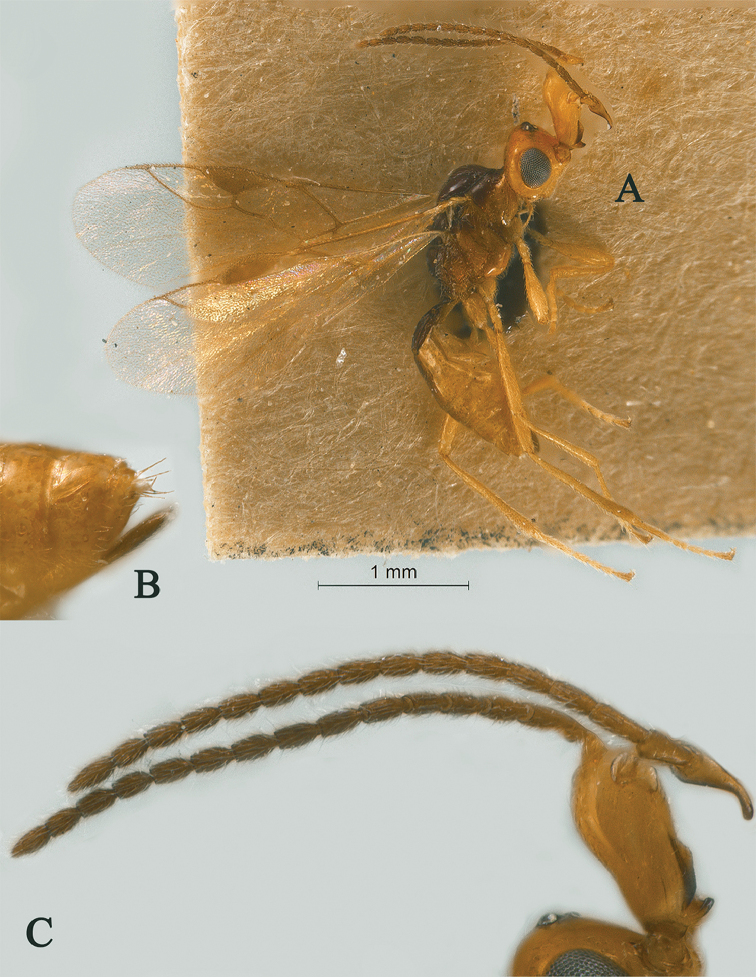
Streblocera (Streblocera) zoroi Li, Chen and van Achterberg, sp. nov., ♀ **A** habitus, lateral aspect **B** ovipositor and its sheath, lateral aspect **C** antenna.

**Figure 28.**
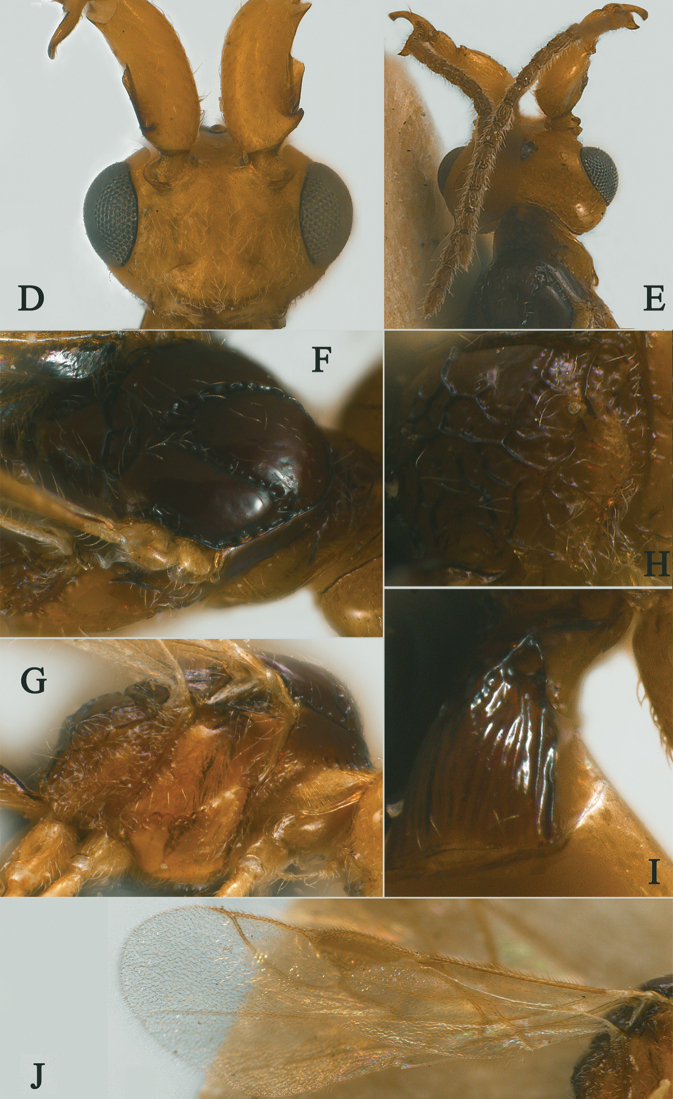
Streblocera (Streblocera) zoroi Li, Chen and van Achterberg, sp. nov., ♀ **D** head, dorsal aspect **E** head, anterior aspect **F** mesosoma, dorsal aspect **G** mesosoma, lateral aspect **H** propodeum, dorsal aspect **I** first metasomal tergites, dorsal aspect **J** fore wing.

Figures 27A–C , 28D–J

#### Type material.

Holotype, ♀, C China, Hubei Province, Shenongjia, Muyu, 8. viii. 1988, Jianquan Yang, average altitude 1200m.

#### Description.

Holotype, ♀, length of antenna 2.2 mm, of fore wing longer than 2.6 mm, and of body 2.6 mm.

*Head.* Antenna with 18 antennomeres and 0.8× as long as body (Fig. 27A); scapus quite stout, slightly curved, weakly expanded and sparsely setose, 2.5× longer than its maximum width, with two horns on basal half: lower horn twisted and upper one shark fin-shaped (Figs 27C, 28D); first and second flagellomeres sparsely setose and modified: first flagellomere with a strong downwards and blunt hook apically, second flagellomere with hook apically and subbasally inserted on first flagellomere (Fig. 27C); first flagellomere 2.0× longer than second flagellomere, first, second and penultimate flagellomere 3.0, 2.0 and 1.8× longer than wide, respectively (Fig. 27C); eye 1.8× longer than temple in dorsal view (Fig. 28E); temples roundly narrowed behind eyes (Fig. 28E); ocelli small, OOL:OD:POL = 67:9:16 (Fig. 28E); frons and vertex smooth; occipital carina nearly complete present, narrowly interrupted medio-dorsally and convex dorsally (Fig. 28E); face 2.5× wider than high, smooth (Fig. 28D); clypeus smooth, narrow than face, strongly convex, 2.7× wider than high (Fig. 28D); dorsal margin of clypeus distinct above level of ventral margin of eye anterior (Fig. 28D); tentorial pits large (Fig. 28D); malar suture narrow, length of malar space 1.3× basal width of mandible (Fig. 28D); mandibles slender (Fig. 28D).

*Mesosoma.* Length of mesosoma 1.9× its height (Fig. 28G); side of pronotum crenulated anteriorly and medially, but largely smooth (Fig. 28G); propleuron smooth and shiny (Fig. 28G); mesopleuron smooth and shiny (Fig. 28G); prepectal medio-ventral carina present (Fig. 28G); episternal scrobe short and wide (Fig. 28G); precoxal sulcus short, wide and crenulate (Fig. 28G); mesonotum sparsely setose, flat, smooth and shiny (Fig. 28F); notauli narrow, posteriorly crenulated; mesoscutum sparsely setose, flattened (Fig. 28F); scutellar sulcus wide and smooth with one distinct crenula (Fig. 28F); scutellum flat, smooth (Fig. 28F); metapleuron largely rugose (Fig. 28G); propodeum with short basal carina, largely rugose and, pentagon-shaped median area dorsally, laterally largely smooth (Fig. 28H).

*Wings.* Fore wing (Fig. 28J): vein 1-SR+M absent; vein 1-R1 0.7× as long as pterostigma; vein SR1+3-SR curved; r:2-SR = 2:87; vein r issued slightly behind middle of pterostigma; vein m-cu cross vein 2-SR; vein cu-a nearly as long as vein 1-CU1 and postfurcal.

*Legs.* Fore leg: tibia 2.9× longer than coxa, 0.8× as long as femur, and femur flat, 3.6× longer than wide; middle leg: tibia 3.7× longer than coxa, 0.9× as long as femur; hind leg: tibia 3.5× longer than coxa, 1.1× longer than femur; hind coxa smooth, 1.8× longer than wide; hind femur, tibia and basitarsus 8.2, 11.6 and 6.6× longer than wide, respectively; hind basitarsus 0.4× as long as tibia; hind fourth tarsal segment 0.7× as long as fifth tarsal segment.

*Metasoma.* First tergite quite robust, 1.3× longer than its apical width, and apical width 2.3× its minimum width, with large dorsope and laterope basally (Fig. 28I); first tergite basally smooth, finely striate laterally (Fig. 28I); following tergites smooth and shiny; ovipositor sheath and ovipositor short and curved upwards (Fig. 27B).

*Colour.* Yellowish brown to dark brown; palpi pale and legs yellowish brown; head, ovipositor sheath and ovipositor brown; antenna brown, but its basal two segments yellowish brown; wing membrane slightly infuscate, pterostigma and veins brown; body dorsally dark brown.

#### Remarks.

This new species is distinguished from related species by its combination of 18 antennomeres, scapus with two specialized horns, first and second flagellomere modified and occipital carina narrowly interrupted medio-dorsally.

#### Biology.

Unknown.

#### Distribution.

Palaearctic: China (Hubei).

#### Etymology.

The species is named after the virtual character Roronoa Zoro, who is a great swordsman in the Japanese animation “One Piece”. The scapus and the first flagellomere of the new species form three catch structures similar to the three swords of Zoro.

### Streblocera (Villocera) villosa

Taxon classificationAnimaliaHymenopteraBraconidae

Papp, 1985

00AA31AF-9255-52DE-9CFE-827D1145804C

Figures 29A–D

Streblocera villosa Papp, 1985: 352.
Streblocera (Cosmophoridia) villosa ; Chou, 1990: 106; Chao, 1993: 68.
Streblocera guizhouensis You and Lou, 1993: 216. Synonymised by Chen and van Achterberg, 1997: 124.
Streblocera (Villosa) villosa ; Chen and van Achterberg, 1997: 124.

#### Material.

1♀, SE China, Fujian Province, Mt Wuyi, Huanggan, 1.viii.1998, Zhi-shan Wu, average altitude 2000m; 1♀, SE China, Fujian Province, Mt Wuyi, Huanggan, 6.vii.1988, Bao-bin Guan, average altitude 2000m; 1♀, SE China, Fujian Province, Mt Wuyi, Tongmu, 9.viii.1988, Jian-hua Ge, average altitude 1400m; 1♀, SE China, Fujian Province, Mt Wuyi, Tongmu, 21.vii.1988, Jian-hua Ge, average altitude 1400m; 1♀, C China, Hubei Province, Shennongjia, Yangri, 27.vii. 1988, Juchang Huang, average altitude 500m; 1♀, C China, Hubei Province, Shennongjia, Muyu, 5. viii. 1988, Li-qin Zhang, average altitude 1200m; 1♀, C China, Hubei Province, Shennongjia, Muyu, 9. viii. 1988, Li-qin Zhang, average altitude 1200m; 1♀, C China, Hubei Province, Shennongjia, Hongping, 18.viii.1988, Li-qin Zhang, average altitude 2000m.

#### Biology.

Attracted to light (Ku, 1997).

#### Distribution.

Oriental: China (Fujian, Guizhou, Taiwan and Zhejiang) and Palaearctic: China (Hubei), Korea.

**Figure 29.**
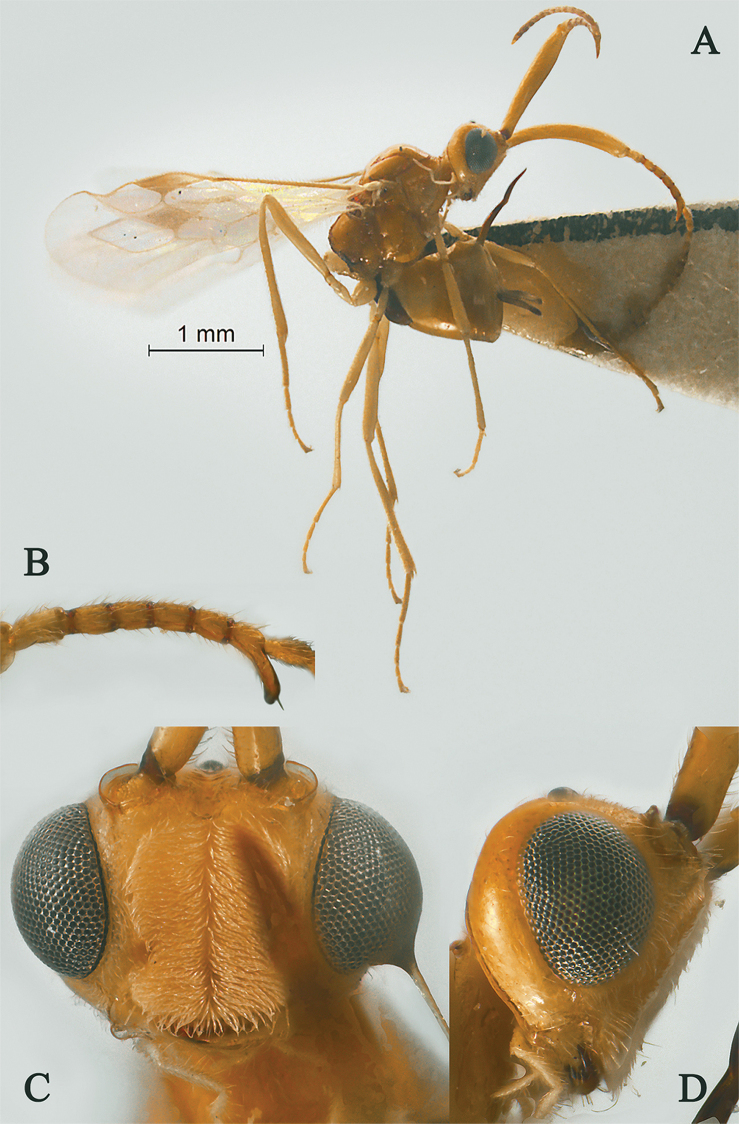
*Streblocera villosa* Papp, 1985, ♀ **A** habitus, lateral aspect **B** first to fifth flagellomeres **C** head, anterior aspect **D** head, lateral aspect.

## Supplementary Material

XML Treatment for Streblocera

XML Treatment for Streblocera (Cosmophoridia) flaviceps

XML Treatment for Streblocera (Eutanycerus) carinifera

XML Treatment for Streblocera (Eutanycerus) chaoi

XML Treatment for Streblocera (Eutanycerus) cornis

XML Treatment for Streblocera (Eutanycerus) hsiufui

XML Treatment for Streblocera (Eutanycerus) laterostriata

XML Treatment for Streblocera (Eutanycerus) lienhuachihensis

XML Treatment for Streblocera (Eutanycerus) okadai

XML Treatment for Streblocera (Eutanycerus) opima

XML Treatment for Streblocera (Eutanycerus) sungkangensis

XML Treatment for Streblocera (Eutanycerus) tsuifengensis

XML Treatment for Streblocera (Eutanycerus) uncifera

XML Treatment for Streblocera (Streblocera) emarginata

XML Treatment for Streblocera (Streblocera) interrupta

XML Treatment for Streblocera (Streblocera) jezoensis

XML Treatment for Streblocera (Streblocera) spasskensis

XML Treatment for Streblocera (Streblocera) stigenbergae

XML Treatment for Streblocera (Streblocera) tayulingensis

XML Treatment for Streblocera (Streblocera) trullifera

XML Treatment for Streblocera (Streblocera) zoroi

XML Treatment for Streblocera (Villocera) villosa
